# Does standard cosmology really predict the cosmic microwave background?

**DOI:** 10.12688/f1000research.22432.5

**Published:** 2021-02-19

**Authors:** Hartmut Traunmüller

**Affiliations:** 1 Department of Linguistics, Stockholm University, Stockholm, SE-106 91, Sweden

**Keywords:** cosmic background radiation, cosmology theory, concordance cosmology, big bang cosmology

## Abstract

In standard Big Bang cosmology, the universe expanded from a very dense, hot and opaque initial state. The light that was last scattered about 380,000 years later, when the universe had become transparent, has been redshifted and is now seen as thermal radiation with a temperature of 2.7 K, the cosmic microwave background (CMB). However, since light escapes faster than matter can move, it is prudent to ask how we, made of matter from this very source, can still see the light. In order for this to be possible, the light must take a return path of the right length. A curved return path is possible in spatially closed, balloon-like models, but in standard cosmology, the universe is “flat” rather than balloon-like, and it lacks a boundary surface that might function as a reflector. Under these premises, radiation that once filled the universe homogeneously cannot do so permanently after expansion, and we cannot see the last scattering event. It is shown that the traditional calculation of the CMB temperature is inappropriate and that light emitted by any source inside the Big Bang universe earlier than half its “conformal age” can only become visible to us via a return path. Although often advanced as the best evidence for a hot Big Bang, the CMB actually tells against a formerly smaller universe and so do also distant galaxies.

## Introduction

In 1964, Penzias & Wilson (1965) serendipitously discovered the cosmic microwave background (CMB), a thermal radiation with a temperature of 2.7 K. Prior to this, the presence of a cosmic heat bath with a temperature of a few K had already been conjectured by several researchers on various grounds unrelated to the Big Bang ( Assis & Neves, 1995). Based on absorption lines of interstellar CN-molecules, McKellar (1940) had suggested a maximum temperature of interstellar space of no more than 2.7 K. Alpher & Herman (1948) and Alpher *et al.* (1967), who were contemplating thermonuclear reactions in the expanding universe (for historical perspectives see Naselsky *et al.* (2006) and Alpher (2012), expected a thermal radiation with about 5 K as a residual of a hot Big Bang. In this, they built on Tolman’s studies ( Tolman, 1931; Tolman, 1934) of model universes filled with blackbody radiation as a thermodynamic fluid, so that “ *The model of the expanding universe with which we deal, then, is one containing a homogeneous, isotropic mixture of matter and blackbody radiation*” ( Alpher & Herman, 1975). They did not really discuss and clarify under which conditions such a state is sustainable in Big Bang models.

When Penzias & Wilson (1965) were bothered by the presence of unexpected radiation, another group of scientists ( Dicke *et al.*, 1965) did expect it in a hot Big Bang model and was developing an experiment in order to measure it. After asking whether the universe could have been filled with black-body radiation from its possible high-temperature state, they say “ *If so, it is important to notice that as the universe expands the cosmological redshift would serve to adiabatically cool the radiation, while preserving the thermal character. The radiation temperature would vary inversely as the expansion parameter (radius) of the universe.*” This is also what Tolman (1934) said.

Dicke *et al.* (1965) were initially in favor of a model in which the universe expands, slows down and contracts to a minimal size (not necessarily a singularity), for a new cycle to begin, but they concluded that “ *with the assumption of general relativity and a primordial temperature consistent with the present 3.5°K, we are forced to adopt an open space, with very low density.*” ( Dicke *et al.*, 1965). They had expected the temperature to exceed 30 K in a closed space.

In subsequent Big Bang models, the universe expanded from a very dense and opaque initial state in which it was filled with a hot and dense plasma consisting of protons, electrons and photons colliding with these. When the plasma had cooled sufficiently by the expansion of the universe, electrons and protons combined into H atoms. This event is still referred to as “recombination”, although cyclic models had lost support in the late 1990s, when an accelerated expansion suggested itself (within the Big Bang paradigm) in the redshift-magnitude relation of supernovae ( Perlmutter, 2012; Riess, 2012; Schmidt, 2012) instead of an expected decelerated one. Only after recombination and decoupling, when the charged particles had been neutralized, the photons could move freely.

It is now commonly estimated that the universe became transparent about 380,000 years after the Big Bang ( Smoot, 2007), when it had cooled to about 3000 K. The thermal radiation is said to have been emitted from a “last scattering surface” (LSS) and to have retained its blackbody spectrum because it expanded adiabatically. Due to the ever continuing expansion, which uses to be ascribed to “space”, the light waves were stretched and their energy density decreased. The wavelength at which the radiation is strongest, which according to Wien’s displacement law is inversely proportional to temperature, would have become roughly 1100 times longer since the radiation was emitted ( Bennet *et al.*, 2003), while the temperature decreased to the present 2.7 K. Since the 1970s, the presence of this radiation has routinely been advanced as the strongest piece of evidence for a hot Big Bang.

The idea that the CMB comes directly, although redshifted, from a last scattering surface emerged only after 1965. It is not clear how the early followers of Tolman (1934) thought about this, but it requires normally a confinement in order to keep blackbody radiation within a region, and the questions of what constitutes or substitutes the confinement of an expanding universe and which difference the motion or absence of a boundary surface would make were not treated critically. The problem we are concerned with here arose at the latest when these questions were still not treated critically when the assumption of a directly viewed LSS had made them crucial.

## The problem

If one considers the following question, one can easily see that Big Bang cosmology requires the universe to be suitably confined or curved in order for radiation from the LSS to become visible at all.

If the CMB originated at the last scattering surface and all matter originated within the region enclosed by this surface, while light escaped from there at *c*, maintaining this velocity for eons, and the matter of which we consist left the same region more slowly, then, how can it be that we can see the light?

In order to see an event, the observer needs to be in a place where the light from the event has not yet passed, but with the stated premises, we cannot reasonably be ahead of the light. The ‘flash’ of light from the LSS had a substantial duration, but it must have passed our place very long ago. Now, it could only become visible at our place if the light had been reflected back to us or taken a curved return path of the right length. In a model, this needs to be specified. Before turning to the standard model, which will be shown to be inconsistent, let us first consider a non-reflective “flat” model and then briefly also reflective versions and a positively curved model.

*Model 1.* In a non-reflective flat Big Bang model (curvature 0), light will escape from the expanding material universe and proceed farther at velocity *c.* The material universe will be surrounded by an expanding empty region inside a spherical shell that contains radiation, perhaps also cosmic rays, but no ordinary matter. In such a universe, the conditions assumed by Tolman (1931); Tolman (1934) and presupposed by his followers are not permanently retained after last scattering. However, the belief that radiation from a past epoch, named “relic radiation” or “residual radiation”, could permanently fill the whole volume of an expanding, formerly smaller universe even in the absence of a reflective boundary surface or a suitable “curvature” was inherent in the reasoning by Alpher & Herman (1948); Alpher *et al.* (1967) and Dicke *et al*. (1965), and it has remained so in the more recent literature, e.g. Peebles *et al.* (1991) and Peebles (1993). Alpher & Herman (1975) described their expanding universe in retrospect as “ *one containing a homogeneous, isotropic mixture of matter and blackbody radiation*”. This can and should be read as a warning against uncritical adoption, since the authors did not reason about how such a state could maintain itself over time, given the speed difference between radiation and matter. Dicke *et al*. (1965) stated that “ *The radiation temperature would vary inversely as the expansion parameter (radius) of the universe*”. Their calculation presupposes the radiation to fill their expanding universe permanently. Likewise, Peebles *et al*. (1991) wrote: “ *In the standard model, … space was (and is) filled with black-body radiation, the cosmic background radiation*”, but the “ *(and is)*” qualifies as a non-sequitur. Correctly and transparently reasoned, radiation from a past epoch fills, at each instant, only the volume that is traversed by the rays or “future light cone” from that epoch.

**Figure 1.**
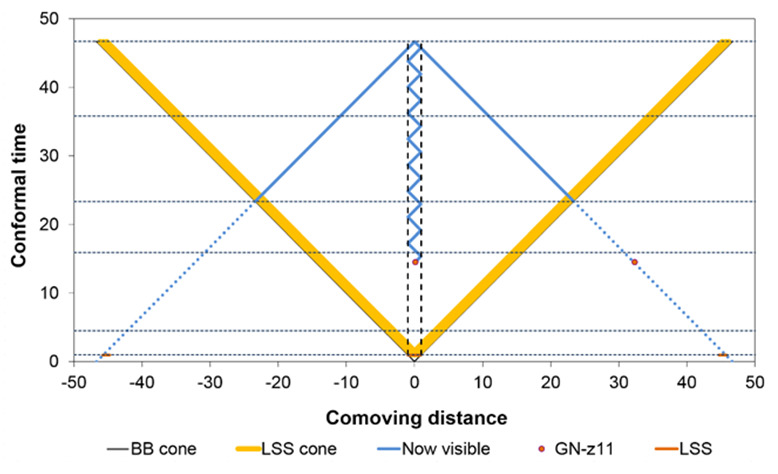
Spacetime diagram of a flat Big Bang universe. Abscissa: comoving distance χ in Glyr. Ordinate: conformal time η in Gyr. V-like golden band: the future light cone of the last scattering surface (LSS, the red horizontal dash close to the zero-point, visible directly only from within the golden band). Blue Λ-like trace: our past light cone – we are located at its peak, not in the golden band. The region beyond the golden band (dotted extension of the blue trace) has not come into existence. In standard cosmology, the galaxy GN-z11 and a fictitious LSS are placed in this region nevertheless (the latter at *χ ≈* ±46 Glyr). Between the dashed vertical lines: a confined universe that co-expands with the material universe (co-moving diameter constant and equal to that of the LSS, mentioned under model 2). So confined, the LSS remains permanently visible. The place of GN-z11 in this model and a zigzag path to us via 17 reflections is also shown. Dotted horizontal lines: see Table 1. Last scattering at *η ≈* 0.95 Gyr, *t ≈* 0.38 Myr; last visibility of the LSS and last blackbody conditions at *η ≈* 1.9 Gyr, *t ≈* 1.95 Myr.

For an origin at the LSS and no reflection, this volume is represented by the golden V-shaped band in Figure 1. The band stands for a radiation-filled shell whose thickness remains, in comoving units, constant and equal to the diameter of the LSS. The shell surrounds an expanding volume that contains no such radiation. In such a universe, the LSS will no longer be visible to anybody who has moved at *v* << *c* when more time has passed than what light needed for crossing the universe just after it had become transparent (the vertical width of the golden bands in Figure 1). The actual CMB we see now thus could not possibly have originated there.

Model 1 is clearly incompatible with the assumption that the universe is filled with a homogeneous mixture of matter and blackbody radiation. In order to find out whether the homogeneity assumption or the Big Bang model should be rejected, it is most persuasive to consider the space the model predicts to be filled with galaxies. This space is somewhat larger than the co-expanding region between the pair of dashed vertical lines in Figure 1, but definitely smaller than the universe, which is delimited by the golden V-shaped band. Since we observe galaxies even beyond this band ( Chambers *et al.*, 1990; Oesch *et al.*, 2016), the model is falsified even without considering the CMB, while the observed properties of the latter corroborate the homogeneity assumption.

*Model 2.* In a flat Big Bang universe that is surrounded by a boundary surface, light can be reflected there. Complete reflection occurs if the impedance of space becomes infinite (or zero) there. If space just loses its existence at an “edge”, the impedance becomes undefined, which is problematic, but the location of the reflective surface is also problematic. In order for the CMB to become visible, the reflection must occur at a certain distance from us, within the future light cone of the LSS. If the reflection occurred at a constant distance from us, this could work in our epoch, but the CMB would not have been visible between our epoch and the time when the direct view of the LSS was lost. If the reflection formerly occurred at a smaller distance, the CMB may have been visible then, but this would have blocked any later view from a larger distance. An elaborate model that avoids this problem and/or describes a view via repetitive reflections at opposite surfaces does not appear to have been proposed.

The present standard model is in some respects equivalent to model 2. In it, the expansion is described by the scale factor *a(t)* = (1 + *z*) ^-1^, which is applied to co-expanding structures in three dimensions and also to the dimension of time, while it is disregarded that radiation not only expands in these four dimensions but also escapes from its origin at *c* and so disappears from direct view, remaining within the golden band in Figure 1. This traditional disregard is an embarrassing blunder.

The disregard would be justified if and as long as the radiation lost from a region was balanced by an equal amount gained from outside. The conditions for this to happen have traditionally been assumed to be met, but this has apparently never been analyzed critically. In a Big Bang universe it is fairly clear from Figure 1 that radiation is lost from a co-expanding region by propagating forward within the golden band while nothing can be gained from outside the universe.

The disregard would also be justified if the material universe was surrounded by a reflective “firmament” whose diameter also expanded at *a(t).* This diameter would, then, remain constant in units of comoving distance, which is a distance measure in which the expansion of the universe has been factored out (consider the dashed vertical lines in Figure 1). If the enclosed space in these units was as large as the LSS, it would indeed remain homogeneously filled with reflected radiation, and the CMB would evolve as traditionally assumed and taught, e.g., in Chapter 6 of Peebles’ (1993) authoritative textbook. However, such a reflective firmament is for good reasons not specified in standard cosmology. It would be incompatible with the cosmological principle even in its imperfect form (which already allows violation in the dimension of time). It remains also unclear how matter that hits the firmament would interact with it.

*Model 3.* In a positively curved Big Bang model (curvature +1), which, reduced by one dimension, can be imagined as the surface of an inflating balloon, the LSS could be visible because these models allow a return path of light. This visibility can be expected to evolve with the expansion factor of the universe from continuous to periodic before finally being lost. Here, we shall not delve into the question under which premises it could be permanent or be lost entirely, because it would require assumptions that are not made in standard cosmology. Instead, when analyses of high-resolution maps of the CMB were found to be compatible with a flat universe ( Davis *et al.*, 2007; de Bernardis *et al.*, 2000) rather than with a positively curved one, the flat universe became adopted as the standard. This flatness came unpredicted and posed a “coincidence problem” ( Debono & Smoot, 2016). Recently, based on CMB data from the Planck mission, a positive curvature has been argued for ( Di Valentino *et al.*, 2019), but this is not a feature of the present standard model.

*Model 4.* In the present standard model ( Ryden, 2017; Smoot, 2007), a “cosmogonic” flat and non-reflective Big Bang model (model 1), in which the universe expanded out of a singularity in spacetime, developed as summarized in the *Introduction*, and is now highly non-homogeneous, as described under *Model 1*, is supplemented with a model that has its origin in the otherwise reasonable but contrary assumption that the universe is, at large, homogeneously filled with matter and blackbody radiation.

The fact that it includes a Big Bang model does not mean that the present standard model remains a Big Bang model when its supplement is invoked. In a proper Big Bang model, there exists nothing at all (not even a physical vacuum) below its future light cone, i.e., below the golden V-shaped band in Figure 1. This is not so in the “standard model”, whose supplement contradictorily presupposes matter to exist in this region.

The homogeneity assumption is drastically incompatible with a Big Bang in flat space, in which radiation from past events, such as from last scattering, cannot fail to separate ever more from the material content of the universe. Neither matter nor radiation can remain homogeneously distributed in the expanding universe. There is no such problem with the isotropy that is also postulated. It has remained unnoticed or at least untold or obfuscated that the adoption of the homogeneity assumption together with a Big Bang process results in a self-contradiction. It appears, preposterously, to be assumed that the homogeneous universe was already as large as it is now, or even infinite, at the time at which it is also assumed to have been much smaller or even to have emerged out of a point-like singularity.

In a model that is slightly less obviously untenable, the universe has always had at least its present size, while time arose 13.7 Gyr ago. The radiation sources that were visible shortly after time onset were all cosmically nearby. As time passed on, the span of distances at which sources could be seen became successively wider. Events that occurred shortly after time onset were visible ever since. This makes the attributes “Expanding View” and “chronogonic” adequate for this model. I had to coin these attributes myself, inspired by correspondence with Edvard Mörtsell and Barbara Ryden. In the literature, the supplementary model uses to be invoked informally, without being named. Together with the common but misleading practice of referring to the entire standard model as a Big Bang model, this dampens the awareness about the presence and contradictoriness of the supplement.

In the present standard model, the CMB radiation density is still calculated in the traditional manner as if the Big Bang universe, whose comoving radius was ≈ 0.95 Gly when it became transparent, was filled with a photon gas within an imaginary box whose volume *V* expands at the same rate as the material universe, so that *V* ∝ *a*( *t*) ^3^ ( Ryden, 2017, section 2.5). The number density of photons would thus remain the same in comoving coordinates. This is in its outcome essentially the same as if the material universe was surrounded by a reflective sphere that co-expanded with the LSS, as in model 2. If the calculations are done as if the imaginary box was present although it is actually absent, then the blunder mentioned in the second passage under *Model 2* is committed: in the absence of a confinement, the radiation cannot fail to escape from this region at *c* (within the golden V in Figure 1). Since this is missed in Ryden’s description, the model is flawed at this point, but before being supplemented, it is still a Big Bang model in which nothing at all exists below the golden V. As soon as one follows Tolman (1934) and assumes that the considered volume gains from its surroundings exactly as much as it loses to them, one defies the Big Bang model already at this point, because nothing can be gained from its non-existent (or at least empty) surroundings.

In Figure 1, the apparent places of origin of the CMB, which suggest a fictitious LSS, are maximally remote, in comoving distance about ±45.7 Glyr farther away from the original LSS, at which the temperature is calculated to have been 3000 K at decoupling, i.e., at *t* = 380 kyr. In terms of comoving distance, the extension of this surface had then already grown to almost ±1 Glyr, but no more than that. Note that the use of ordinary, unexpanded coordinates would make the place-discrepancy much smaller, but it would not make any difference to what is inside and outside the Big Bang universe.

The apparent origin of the CMB in a maximally remote spherical surface or shell around our position (see Figure 8.4 in Ryden, 2017) is only compatible with the Expanding View model. A flat Big Bang universe in which no reflection occurs contains no sufficiently remote points of origin. The supplementary model takes care of this by turning the Big Bang universe inside out. Since the original LSS in the unsupplemented Big Bang model is still needed for calculating the properties of the CMB, standard cosmology operates with two drastically different locations of the same last scattering event, and this is irrational.

By the way, simply turning the Big Bang model inside out does not invalidate the initial statements under “ *The problem*”. Even if this is done, which is a drastic error, it still needs to be considered that light propagates from the LSS faster than the constituent matter of an observer can have moved. This precludes a common place of origin for matter and the CMB also at the periphery of the visible universe.

It is not either possible to replace the Big Bang model entirely by the Expanding View model, because the latter does not predict the properties of the CMB based on its own premises – not even the existence of a homogeneous LSS and that of a cosmic redshift. The CMB and the cosmic redshift might have other origins and reasons, but we are here only concerned with standard cosmology.

While CMB photons may actually require 13.7 Gyr to reach us from their source, and the Universe may well be flat and infinite, a flat and reflection-free Big Bang universe does not provide the spacetime that would be necessary in order to accommodate a ray (a null-geodesic) of the corresponding length. If a ray of this length is to end at us, it must have its origin outside the Big Bang universe. This may well be so, but if this is accepted, as it is in the Expanding View model, then, the very idea of a Big Bang is untenable and, if reason rules, thereby already rejected. It is then irrational to calculate the properties of the CMB on the basis of its origin at a LSS inside a Big Bang universe and simultaneously to admit its origin at a maximally remote place outside the said universe, where the conditions are very different if ascertainable at all. The custom of pretending that processes such as “last scattering”, “decoupling” etc., would occur also in a chronogonic universe, is deceptive.

The zero-point of time in the chronogonic Expanding View model is an intellectual relic from Big Bang models. In these, it is the time at which there was a singularity in space. If this singularity in space is removed, as it is in the chronogonic model, then any zero-point in time will be arbitrary and must be physically inconsequential. This implies that the radiation density in the universe cannot have been higher at points in time that were closer to the zero-point than we are now. Thus, in the cosmogonic model, the LSS existed but cannot be seen by us, while in the chronogonic model it never existed at all. If this is to be amended, we have to go for a model that is neither cosmogonic nor chronogonic, but in which the universe, if it is homogeneous at the largest scale, always can have shown the same appearance at this scale.

Figure 1 illustrates the relevance of the problem to other observables than the CMB as well: in a flat geometry, our direct view is limited to events that happened after the universe had attained half its present age in conformal time (at *η* ≈ 23.35 Gyr). This corresponds to *t* ≈ 1.7 Gyr, scale factor *a*( *t*) ≈ 0.21 and redshift *z* ≈ 3.78. It is noted as “conformal halftime” in Table 1. In order for earlier events to be seen, Big Bang cosmology requires light to take a straight or curved forward and return path. This appears to have gone unnoticed by observers of distant galaxies. About GN-z11, with redshift *z* = 11.09, it is reported that “ *This indicates that this galaxy lies at only ~400 Myr after the Big Bang*” ( Oesch *et al.*, 2016), at *a(t) ≈* 0.083. This actually puts the galaxy, shown in Figure 1, far beyond the future light cone of the Big Bang. If anything exists in this spacetime region, it cannot have arrived there from the presumed ultimate origin of matter. The first galaxy that, with *z* = 3.8, was too far away to be seen directly in a Big Bang universe had been observed already in 1987 ( Chambers *et al.*, 1990). If galaxies at *z* > 4 cannot even be located within such a universe, it is no longer a surprise that they do not show the evolution they should according to the hierarchical merging paradigm that has become part of concordance cosmology ( Steinhardt *et al.*, 2016).

**Table 1.** Values of scale factor *a*, redshift *z* and age *t* of the universe, listed for conformal times *η* represented by dotted horizontal lines in Figure 1.

Conformal time *η* (Gyr)	*a*	*z*	*t* (Gyr)	Notes
46.7	1	0	13.7	Now
35.8	0.5	1	5.95	
23.35	0.21	3.76	1.70	Conformal halftime
15.9	0.1	9	0.56	
4.5	0.01	99	0.017	
1.0	0.001	999	0.00044	

Values based on 5-year WMAP data and ΛCDM model computed using WolframAlpha ^®^.

In stark contrast to what is traditionally claimed, the CMB actually tells against a formerly smaller universe and so do the most distant galaxies. The visibility of these has not been reconciled with the idea of a Big Bang. The related attempt to do so has led to a confused use of models that are incompatible with each other. The need for invoking the Expanding View model would disappear if we actually saw mirror images [as in model 2], but in order for galaxies to be seen in this way and the actual isotropy of the CMB to be obtained, the reflector would need to be of all too spectacular stability and flatness - like that required in a telescope of giga-lightyears in length.

## Discussion

Because of the inherent inconsistency of the standard ΛCDM concordance cosmology, here represented by model 4, it does not come as a surprise that “ *misconceptions and confusions have long been common in papers on cosmology, also in many by renowned authors*”, as reported by Davis & Lineweaver (2004). These authors deserve credit for having paid attention to those. However, they did not either notice that early events cannot be seen directly. In proceeding without considering reflections (last passage of their section 3.3), they mistook the intersection between our past light cone and the future light cone of the LSS [where a reflection would need to occur] for “ *the points from which the CMB was emitted*” ( Davis & Lineweaver, 2004, p. 101). Although this is not yet beyond the particle horizon of the Big Bang, it would still be off target by half as much as model 4. The confusion arose by equating this particle horizon with the surface of last scattering, which the authors refer to as “ *our effective particle horizon*” ( Davis & Lineweaver, 2004). It also disagrees with the caption of their Figure 1, which presupposes model 4 as such.

When Tolman (1931) considered “ *the highly idealized model of a non-static universe filled with black-body radiation as a thermodynamic fluid*”, he did not discuss the implications of the large size of the universe and the possible absence of a reflective confinement or its equivalent. It deserves to be noted that the time required for cavity radiation to attain a desired degree of homogeneity (after a sufficient number of reflections) increases in proportion to the linear size of the cavity. In a Big Bang universe, this will even with modest demands take much longer than its age. If there is no boundary surface other than one that recedes at *c*, we have seen that any old radiation will eventually disappear from view. In a flat and non-reflective Big Bang universe [model 1 above and its equivalent in model 4 before being supplemented], this must happen to the radiation from the original LSS, which, thus, cannot remain visible. The CMB must have a different source, whose identification exceeds the scope of this article.

It is futile to consider whether the cosmic inflation theory ( Guth, 1981) might solve the homogeneity problem, because the process this theory postulates is terminated long before recombination. In the present article, the homogeneity at the stage of recombination in a Big Bang universe is not put into question. Instead, it is pointed out that homogeneity will be lost *thereafter*, irrespective of anything that might happen before.

While the irrationality of the assumption about the visibility of radiation from a past epoch in a Big Bang universe, which was disclosed in *The problem*, can be clearly seen in a spacetime diagram such as Figure 1, it may be missed if the ordinary coordinates of time and distance are used, especially if a past light cone is shown (in these coordinates shaped like an avocado seed) that continues below *t* = 1.7 Gyr down to the origin, while it is not made evident that the region it traverses there lies outside the Big Bang universe. For examples see the “avocado seeds” in Davis & Lineweaver (2004), more detailed in Whittle and without any scale under “Manipulating Space-Time Diagrams” in Wright.

The fact that the irrationality has remained unnoticed by professionals is an instance of the ordinary uncritical passing down of human culture, of languages, myths, etc. from generation to generation. In this wider cultural context, science stands out as an exceptional, more critical endeavor that requires practitioners not simply to accept and adopt what they were taught, but to check the relevant assumptions and doctrines for consistency and tenability and to recheck them when premises and/or relevant knowledge change. This may sometimes fail to happen, especially in cases like this, where the presence of an inconsistency became potentially clear only gradually, here after 1965, when a teaching practice had already established itself since Tolman (1934). This practice appears to have prevented the disclosure of the irrationality, which would likely have become obvious after a fresh look at the facts. It is in line with this and with Lakatos’ (1976) analysis of research programs that the rejection of the idea of a Big Bang has been blocked in model 4, although the evidence that requires the rejection has been accepted. Blockage of this kind tends to foster more or less absurd speculation. While scientific journals often tolerate speculative ideas like “inflation” and the “multiverse”, which have been left out of consideration here, it is unfortunate that most of them refuse through prejudice to publish any paper that discredits the “hard core” ( Lakatos, 1976) of the currently accepted doctrine within their field from inside. For editors, it is rational to reject such papers right away: these might threat their reputation if later shown to be erroneous. Also for reviewers who lack a critical attitude against the established practice and doctrine, it is a priori inconceivable that the whole community of well-educated professionals, here mainstream cosmologists, could have made the same cardinal blunder. This holds also in cases like this one, in which the presence of at least one inconsistency is obvious to the uncommitted.

Although the deficiencies disclosed here can be judged as completely unacceptable, other ones need to be addressed as well ( López-Corredoira, 2017; Merritt, 2017; Spergel, 2015; Traunmüller, 2014; Traunmüller, 2018). Just consider that both Λ (dark energy) and CDM (cold dark matter) have remained in the imaginary realm and so merely represent mythical factors or immunizing tactics (also called “conventionalist stratagems”) that protect a doctrine from empirical falsification ( Merritt, 2017). Approaches that rely on such factors are excessively speculative, but inconsistencies such as the two revealed here must be desisted from in any discipline that is meant to qualify as rational. Within standard Big Bang cosmology, there is at least one additional inconsistency that is similarly serious. It is well-known that in this cosmology, any coherent and gravitationally bound objects up to the size of galaxy clusters are exempt from expansion. Only the voids between these clusters are free to expand (Traunmüller, 2018). Under this premise, the matter density within the universe could never have been higher than it uses to be within galaxy clusters – never as high as assumed during the alleged epoch of last scattering. I am not aware of an excuse for this, but suggesting some fancy new physics that might hide inconsistencies is not the preferable way. One should first look for and correct old mistakes that might cause the inconsistencies. One should strive for well-foundedness in the physical principles ( Traunmüller, 2018) rather than merely for a rationalized mythology, but it is, of course, even more fundamental to respect reason at all.

## Data availability

No data are associated with this article.
