# Does standard cosmology really predict the cosmic microwave background?

**DOI:** 10.12688/f1000research.22432.6

**Published:** 2021-09-23

**Authors:** Hartmut Traunmüller

**Affiliations:** 1 Department of Linguistics, Stockholm University, Stockholm, SE-106 91, Sweden

**Keywords:** cosmic background radiation, cosmology theory, concordance cosmology, big bang cosmology

## Abstract

In standard Big Bang cosmology, the universe expanded from a very dense, hot and opaque initial state. The light that was last scattered about 380,000 years later, when the universe had become transparent, has been redshifted and is now seen as thermal radiation with a temperature of 2.7 K, the cosmic microwave background (CMB). However, since light escapes faster than matter can move, it is prudent to ask how we, made of matter from this very source, can still see the light. In order for this to be possible, the light must take a return path of the right length. A curved return path is possible in spatially closed, balloon-like models, but in standard cosmology, the universe is “flat” rather than balloon-like, and it lacks a boundary surface that might function as a reflector. Under these premises, radiation that once filled the universe homogeneously cannot do so permanently after expansion, and we cannot see the last scattering event. It is shown that the traditional calculation of the CMB temperature is inappropriate and that light emitted by any source inside the Big Bang universe earlier than half its “conformal age” can only become visible to us via a return path. Although often advanced as the best evidence for a hot Big Bang, the CMB actually tells against a formerly smaller universe and so do also distant galaxies.

## Introduction

In 1964, Penzias & Wilson (1965) serendipitously discovered the cosmic microwave background (CMB), a thermal radiation with a temperature of 2.7 K. Prior to this, the presence of a cosmic heat bath with a temperature of a few K had already been conjectured by several researchers on various grounds unrelated to the Big Bang ( Assis & Neves, 1995). Based on absorption lines of interstellar CN-molecules, McKellar (1940) had suggested a maximum temperature of interstellar space of no more than 2.7 K. Alpher & Herman (1948) and Alpher *et al.* (1967), who were contemplating thermonuclear reactions in the expanding universe (for historical perspectives see Naselsky *et al.* (2006) and Alpher (2012), expected a thermal radiation with about 5 K as a residual of a hot Big Bang. In this, they built on Tolman’s studies ( Tolman, 1931; Tolman, 1934) of model universes filled with blackbody radiation as a thermodynamic fluid, so that “ *The model of the expanding universe with which we deal, then, is one containing a homogeneous, isotropic mixture of matter and blackbody radiation*” ( Alpher & Herman, 1975). They did not really discuss and clarify under which conditions such a state is sustainable in Big Bang models.

When Penzias & Wilson (1965) were bothered by the presence of unexpected radiation, another group of scientists ( Dicke *et al.*, 1965) did expect it in a hot Big Bang model and was developing an experiment in order to measure it. After asking whether the universe could have been filled with black-body radiation from its possible high-temperature state, they say “ *If so, it is important to notice that as the universe expands the cosmological redshift would serve to adiabatically cool the radiation, while preserving the thermal character. The radiation temperature would vary inversely as the expansion parameter (radius) of the universe.*” This is also what Tolman (1934) said.

Dicke *et al.* (1965) were initially in favor of a model in which the universe expands, slows down and contracts to a minimal size (not necessarily a singularity), for a new cycle to begin, but they concluded that “ *with the assumption of general relativity and a primordial temperature consistent with the present 3.5°K, we are forced to adopt an open space, with very low density.*” ( Dicke *et al.*, 1965). They had expected the temperature to exceed 30 K in a closed space.

In subsequent Big Bang models, which are based on General Relativity and in which the universe expanded persistently from a very dense and opaque initial state in which it was filled with a hot and dense plasma consisting of protons, electrons and photons colliding with these. This is often referred to as the “primeval” or “primordial” fireball. When the plasma had cooled sufficiently by the expansion of the universe, electrons and protons combined into H atoms. This event is still referred to as “recombination”, although cyclic models had lost support in the late 1990s, when an accelerated expansion suggested itself (within the Big Bang paradigm) in the redshift-magnitude relation of supernovae ( Perlmutter, 2012; Riess, 2012; Schmidt, 2012) instead of an expected decelerated one. Only after recombination and decoupling, when the charged particles had been neutralized, the photons could move freely.

It is now commonly estimated that the universe became transparent about 380,000 years after the Big Bang ( Smoot, 2007), when it had cooled to about 3000 K. The thermal radiation is said to have been emitted from a “last scattering surface” (LSS) and to have retained its blackbody spectrum because it expanded adiabatically. Due to the ever continuing expansion, which uses to be ascribed to “space”, the light waves were stretched and their energy density decreased. The wavelength at which the radiation is strongest, which according to Wien’s displacement law is inversely proportional to temperature, would have become roughly 1100 times longer since the radiation was emitted ( Bennet *et al.*, 2003), while the temperature decreased to the present 2.7 K. Since the 1970s, the presence of this radiation has routinely been advanced as the strongest piece of evidence for a hot Big Bang.

The idea that the CMB comes directly, although redshifted, from a last scattering surface emerged only after 1965. It is not clear how the early followers of Tolman (1934) thought about this, but it requires normally a confinement in order to keep blackbody radiation within a region, and the questions of what constitutes or substitutes the confinement of an expanding universe and which difference the motion or absence of a boundary surface would make were not treated critically. The problem we are concerned with here arose at the latest when these questions were still not treated critically when the assumption of a directly viewed LSS had made them crucial.

## The problem

If one considers the following question, one can easily see that Big Bang cosmology requires the universe to be suitably confined or curved in order for radiation from the LSS to become visible at all.

If the CMB originated at the last scattering surface and all matter originated within the region enclosed by this surface, while light escaped from there at *c*, maintaining this velocity for eons, and the matter of which we consist left the same region more slowly, then, how can it be that we can see the light?

In order to see an event, the observer needs to be in a place where the light from the event has not yet passed, but with the stated premises, we cannot reasonably be ahead of the light. The ‘flash’ of light from the LSS had a substantial duration, but it must have passed our place very long ago. Now, it could only become visible at our place if the light had been reflected back to us or taken a curved return path of the right length. In a model, this needs to be specified. Before turning to the standard model, which will be shown to be inconsistent, let us first consider a non-reflective “flat” model and then briefly also reflective versions and a positively curved model.

*Model 1.* In a non-reflective flat Big Bang model (curvature 0), light will escape from the expanding material universe and proceed farther at velocity *c.* The material universe will be surrounded by an expanding empty region inside a spherical shell that contains radiation, perhaps also cosmic rays, but no ordinary matter. In such a universe, the conditions assumed by Tolman (1931); Tolman (1934) and presupposed by his followers are not permanently retained after last scattering. However, the belief that radiation from a past epoch, named “relic radiation” or “residual radiation”, could permanently fill the whole volume of an expanding, formerly smaller universe even in the absence of a reflective boundary surface or a suitable “curvature” was inherent in the reasoning by Alpher & Herman (1948); Alpher *et al.* (1967) and Dicke *et al*. (1965), and it has remained so in the more recent literature, e.g. Peebles *et al.* (1991) and Peebles (1993). Alpher & Herman (1975) described their expanding universe in retrospect as “ *one containing a homogeneous, isotropic mixture of matter and blackbody radiation*”. This can and should be read as a warning against uncritical adoption, since the authors did not reason about how such a state could maintain itself over time, given the speed difference between radiation and matter. Dicke *et al*. (1965) stated that “ *The radiation temperature would vary inversely as the expansion parameter (radius) of the universe*”. Their calculation presupposes the radiation to fill their expanding universe permanently. Likewise, Peebles *et al*. (1991) wrote: “ *In the standard model, … space was (and is) filled with black-body radiation, the cosmic background radiation*”, but the “ *(and is)*” qualifies as a non-sequitur. Correctly and transparently reasoned, radiation from a past epoch fills, at each instant, only the volume that is traversed by the rays or “future light cone” from that epoch.

**Figure 1.**
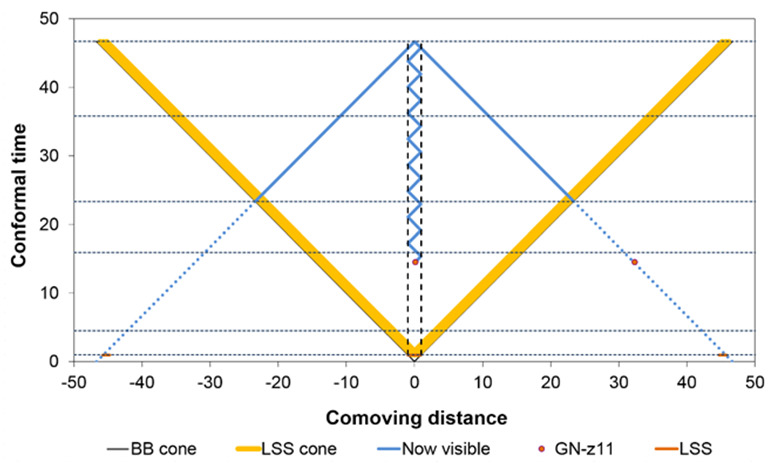
Spacetime diagram of a flat Big Bang universe. Abscissa: comoving distance χ in Glyr. Ordinate: conformal time η in Gyr. V-like golden band: the future light cone of the last scattering surface (LSS, the red horizontal dash close to the zero-point, visible directly only from within the golden band). Blue Λ-like trace: our past light cone – we are located at its peak, not in the golden band. The region beyond the golden band (dotted extension of the blue trace) has not come into existence. In standard cosmology, the galaxy GN-z11 and a fictitious LSS are placed in this region nevertheless (the latter at *χ ≈* ±46 Glyr). Between the dashed vertical lines: a confined universe that co-expands with the material universe (co-moving diameter constant and equal to that of the LSS, mentioned under model 2). So confined, the LSS remains permanently visible. The place of GN-z11 in this model and a zigzag path to us via 17 reflections is also shown. Dotted horizontal lines: see Table 1. Last scattering at *η ≈* 0.95 Gyr, *t ≈* 0.38 Myr; last visibility of the LSS and last blackbody conditions at *η ≈* 1.9 Gyr, *t ≈* 1.95 Myr.

For an origin at the LSS and no reflection, this volume is represented by the golden V-shaped band in Figure 1. The band stands for a radiation-filled shell whose thickness remains, in comoving units, constant and equal to the diameter of the LSS. The shell surrounds an expanding volume that contains no such radiation. In such a universe, the LSS will no longer be visible to anybody who has moved at *v* << *c* when more time has passed than what light needed for crossing the universe just after it had become transparent (the vertical width of the golden bands in Figure 1). The actual CMB we see now thus could not possibly have originated there.

Model 1 is clearly incompatible with the assumption that the universe is filled with a homogeneous mixture of matter and blackbody radiation. In order to find out whether the homogeneity assumption or the Big Bang model should be rejected, it is most persuasive to consider the space the model predicts to be filled with galaxies. This space is somewhat larger than the co-expanding region between the pair of dashed vertical lines in Figure 1, but definitely smaller than the universe, which is delimited by the golden V-shaped band. Since we observe galaxies even beyond this band ( Chambers *et al.*, 1990; Oesch *et al.*, 2016), the model is falsified even without considering the CMB, while the observed properties of the latter corroborate the homogeneity assumption.

*Model 2.* In a flat Big Bang universe that is surrounded by a boundary surface, light can be reflected there. Complete reflection occurs if the impedance of space becomes infinite (or zero) there. If space just loses its existence at an “edge”, the impedance becomes undefined, which is problematic, but the location of the reflective surface is also problematic. In order for the CMB to become visible, the reflection must occur at a certain distance from us, within the future light cone of the LSS. If the reflection occurred at a constant distance from us, this could work in our epoch, but the CMB would not have been visible between our epoch and the time when the direct view of the LSS was lost. If the reflection formerly occurred at a smaller distance, the CMB may have been visible then, but this would have blocked any later view from a larger distance. An elaborate model that avoids this problem and/or describes a view via repetitive reflections at opposite surfaces does not appear to have been proposed.

The present standard model is in some respects equivalent to model 2. In it, the expansion is described by the scale factor *a(t)* = (1 + *z*) ^-1^, which is applied to co-expanding structures in three dimensions and also to the dimension of time, while it is disregarded that radiation not only expands in these four dimensions but also escapes from its origin at *c* and so disappears from direct view, remaining within the golden band in Figure 1. This traditional disregard is an embarrassing blunder.

The disregard would be justified if and as long as the radiation lost from a region was balanced by an equal amount gained from outside. The conditions for this to happen have traditionally been assumed to be met, but this has apparently never been analyzed critically. In a Big Bang universe it is fairly clear from Figure 1 that radiation is lost from a co-expanding region by propagating forward within the golden band while nothing can be gained from outside the universe.

The disregard would also be justified if the material universe was surrounded by a reflective “firmament” whose diameter also expanded at *a(t).* This diameter would, then, remain constant in units of comoving distance, which is a distance measure in which the expansion of the universe has been factored out (consider the dashed vertical lines in Figure 1). If the enclosed space in these units was as large as the LSS, it would indeed remain homogeneously filled with reflected radiation, and the CMB would evolve as traditionally assumed and taught, e.g., in Chapter 6 of Peebles’ (1993) authoritative textbook. However, such a reflective firmament is for good reasons not specified in standard cosmology. It would be incompatible with the cosmological principle even in its imperfect form (which already allows violation in the dimension of time). It remains also unclear how matter that hits the firmament would interact with it.

*Model 3.* In a positively curved Big Bang model (curvature +1), which, reduced by one dimension, can be imagined as the surface of an inflating balloon, the LSS could be visible because these models allow a return path of light. This visibility can be expected to evolve with the expansion factor of the universe from continuous to periodic before finally being lost. Here, we shall not delve into the question under which premises it could be permanent or be lost entirely, because it would require assumptions that are not made in standard cosmology. Instead, when analyses of high-resolution maps of the CMB were found to be compatible with a flat universe ( Davis *et al.*, 2007; de Bernardis *et al.*, 2000) rather than with a positively curved one, the flat universe became adopted as the standard. This flatness came unpredicted and posed a “coincidence problem” ( Debono & Smoot, 2016). Recently, based on CMB data from the Planck mission, a positive curvature has been argued for ( Di Valentino *et al.*, 2019), but this is not a feature of the present standard model.

In present standard cosmology ( Ryden, 2017; Smoot, 2007), a “cosmogonic” flat and non-reflective Big Bang model (model 1), in which the universe expanded out of a singularity in spacetime, is modified for some purposes and, for other purposes, *replaced* by a merely “chronogonic” expanding view model. The said modification results in model 4, while the replacement will be considered under *Model 5*. In model 1, the LSS would be invisible to us. In model 4, neither the visibility of the LSS nor the homogeneity of the radiation from it is lost – but this is due to a blunder. Model 4 is used for describing the alleged development of the properties of the CMB, such as its radiation density and its temperature as *T* ∝ 1/ *a*( *t*) (eq. 6.3 in Peebles, 1993) from 3000 K to 2.7 K. In these calculations, it is considered that the electromagnetic waves after last scattering expand by the factor of 1/ *a*( *t*) in all three spatial dimensions as well as in time. The calculation is done as if the radiation did not really propagate but remained within an expanding spherical region whose comoving diameter of about 1.9 Gly (the length of the red dash close to the origin in Figure 1) remains constant. One speaks of a “relic radiation”, more rarely “relict radiation” or “fossil radiation”. The development is sometimes imagined by assuming that the universe is filled with a photon gas whose volume *V* expands as *V* ∝ *a*( *t*) ^3^ ( Ryden, 2017, section 2.5). In comoving coordinates, the photon gas thus does not expand at all, and the number density of the photons remains constant.

This train of thought can be conveniently referred to as the “relic radiation blunder”. It is easy to see that it is a blunder: After last scattering, photons no longer behave like a gas, whose particles collide randomly with each other, but they propagate unhindered. They will not stay within the said volume, which does not expand fast enough, but they are bound to *escape* at *c* in the V-shaped golden band of Figure 1. They will so remain outside our view and they leave *no* residue behind. The radiation is only visible from spacetime positions within the golden band. Since we are not there and still can see the CMB, its presence requires a different explanation. It may well be a relict of some old radiation. The blunder lies in the way this is presumed to show itself in a Big Bang universe. It reflects the practice of modeling the universe in General Relativity by a uniform expanding fluid, without distinguishing between matter and radiation, and it has been handed down uncritically during the whole history of Big Bang cosmology. By attempting to correct it, one would risk exposing the Big Bang as untenable.

In Figure 1, the pair of dashed vertical lines delimits a volume whose diameter expands in proportion to the scale factor. But in order for radiation to be able to propagate *unhindered* at *c*, the Big Bang universe must expand at *c*. It must, at any chosen point in time (at any vertical position in Figure 1), extend to the outer edges of the V-shaped golden band.

Although marred by an elementary blunder, model 4 is still a Big Bang model in which nothing at all exists below the golden V in Figure 1. As soon as one follows Tolman (1934) and assumes that a considered volume gains from its surroundings exactly the quantity and quality of radiation that it loses to them, one defies the idea of a Big Bang universe already at this point, because the non-existent or at least empty exterior of such a universe offers nothing at all to be gained.

If the relic radiation blunder (model 4) is avoided, it is in any case clear that the CMB does not originate in a primeval fireball. In Big Bang models (like model 1), radiation with this origin would be invisible. In order to be visible at the alleged distance, it would need to originate elsewhere, far outside the primeval fireball.

*Model 5.* The models 1 to 4 attempt to describe how certain aspects of expanding universes evolve over time, given certain physical premises which ‘explain’ the observables. In a cosmogonic Big Bang model, the region located in Figure 1 below the golden V-shaped band does not exist or is, at least, empty. This band represents the “future light cone” that originates when the expanding universe becomes transparent.

Model 5 attempts, instead, to describe where observable objects and events are located along the blue Λ in Figure 1, which represents our “past light cone” in a universe whose spatial extent is not limited in the way it is in a Big Bang model. Model 5 allows things to exist in the region below the golden V, where nothing can exist in Big Bang models. The blue Λ *has been dotted* there. Then there is nothing below the abscissa even in model 5, because it shares an absolute zero point of cosmic time with Big Bang models.

In model 5, time arose 13.8 Gyr ago, while a universe of infinite extent was present already at the onset of time. This universe became gradually visible. The first radiation sources that became visible were all cosmically nearby. As time passed on, the span of distances at which sources could be seen became successively wider, but light emitted 380,000 years after time onset was visible ever since. This makes the attribute “Expanding View” adequate for this model. I coined it myself, inspired by correspondence with Barbara Ryden. In the literature, model 5 uses to be invoked tacitly, without being named. This may dampen the awareness about its deviance and makes it difficult to tell who used it first. The ab initio presence of an infinite or at least a very large universe appears to be an unintentional innovation. Such a universe was not among the alternatives that were considered when the FLRW models were conceived. It is not a Big Bang universe.

In a cosmogonic Big Bang universe, like models 1 and 4, the galaxy GN-z11 cannot be at a comoving distance of about 32 Glyr when only 15 Gyr conformal time had passed (see Figure 1). It would require a superluminal velocity to place anything there if this space existed at all. This position is, however, compatible with the Expanding View model, in which the galaxy may have been at a distance of about 32 Glyr already at the onset of time. In this model, radiation with an origin at the epoch of last scattering is now visible at a comoving distance of about 46 Glyr. An event of last scattering is, however, only predicted by the Expanding Space model, in which it occurs at a comoving distance of less than 1 Glyr. By itself, the Expanding View model does not require a compact initial state.

In “standard cosmology” an Expanding View model is placed over a Big Bang model. In this way, it is now, in effect, taught that, in comoving coordinates, the universe was already as large as it is now, or even infinite (in model 5), at the time at which it was much smaller than now (in models 1 and 4) or even emerged out of a point-like singularity in spacetime. This blatant contradiction arises if distances on our past light cone are calculated without regard to the fact that a cosmogonic expanding universe must have been smaller than it is now when the light from a distant source was emitted. In a flat universe, any light beam that is longer than 23.35 comoving Glyr would reach us from a source outside the Big Bang universe, i.e., from below the golden V in Figure 1. What is, in effect, done when our distance from a radiation source is estimated, is to estimate the time that has passed since the observed radiation was emitted, and to multiply this with *c*. This can result in comoving distances up to 46.7 Glyr. By allowing light beams that are this long, the dotted continuation being added to the blue Λ in Figure 1, the spatial limitation of the Big Bang model is completely removed. Only the temporal one remains. Instead of a “cosmogonic” model, we then have a merely “chronogonic” one. This implies even more absurd initial conditions, which we shall not delve into here.

In comoving coordinates, the places of origin of the CMB suggested by the models 4 and 5 are maximally remote from each other. The one suggested by model 5 is about ±45.7 Glyr farther away from the one in model 4, in which the temperature is calculated to have been 3000 K at decoupling, i.e., at *t* = 380 kyr. In terms of comoving distance, the extension of this surface had then already grown to almost ±1 Glyr, but no more than that. In ordinary, unexpanded coordinates, the place-discrepancy is much smaller than in comoving ones, but the choice of coordinates makes no difference to what is inside and outside the Big Bang universe, and the discrepancy remains the same in relative terms.

The apparent origin of the CMB in a maximally remote spherical surface or shell around our position (see also Figure 8.4 in Ryden, 2017) is only compatible with the Expanding View model. A cosmogonic flat Big Bang universe in which no reflection occurs contains no sufficiently remote points of origin. Since the LSS in model 4 is still used in deriving the properties of the CMB, standard cosmology operates with two drastically different locations and sizes of what is meant to be one and the same radiation source. This defies rationality.

It may be that distance measures that go beyond the scope of Big Bang models lead to a tenable description of the universe. However, accepting this requires rejecting the cosmogonic Big Bang. In this case, the CMB and its homogeneity must have a different origin and reason, but we are here only concerned with standard cosmology.

The absolute zero-point of time in the chronogonic Expanding View model is a relic from Big Bang models. In these, it is the time at which there was a singularity in space. If this singularity in space is removed, as it is in the chronogonic model, then any zero-point in time will be arbitrary and must be physically inconsequential. In a cosmogonic model, the LSS existed but cannot be seen by us, while in the chronogonic model it never existed at all. If this is to be amended, we have to go for a model that is neither cosmogonic nor chronogonic, but in which the universe, if it is homogeneous at the largest scale, always can have shown the same appearance at this scale.

Figure 1 illustrates the relevance of the problem to other observables than the CMB as well: in a flat geometry, our direct view is limited to events that happened after the universe had attained half its present age in conformal time (at *η* ≈ 23.35 Gyr). This corresponds to *t* ≈ 1.7 Gyr, scale factor *a*( *t*) ≈ 0.21 and redshift *z* ≈ 3.78. It is noted as “conformal halftime” in Table 1. In order for earlier events to be seen, Big Bang cosmology requires light to take a straight or curved forward and return path. This appears to have gone unnoticed by observers of distant galaxies. About GN-z11, with redshift *z* = 11.09, it is reported that “ *This indicates that this galaxy lies at only ~400 Myr after the Big Bang*” ( Oesch *et al.*, 2016), at *a(t) ≈* 0.083. This actually puts the galaxy, shown in Figure 1, far beyond the future light cone of the Big Bang. If anything exists in this spacetime region, it cannot have arrived there from the presumed ultimate origin of matter. The first galaxy that, with *z* = 3.8, was too far away to be seen directly in a Big Bang universe had been observed already in 1987 ( Chambers *et al.*, 1990). If galaxies at *z* > 4 cannot even be located within such a universe, it is no longer a surprise that they do not show the evolution they should according to the hierarchical merging paradigm that has become part of concordance cosmology ( Steinhardt *et al.*, 2016).

**Table 1.** Values of scale factor *a*, redshift *z* and age *t* of the universe, listed for conformal times *η* represented by dotted horizontal lines in Figure 1.

Conformal time *η* (Gyr)	*a*	*z*	*t* (Gyr)	Notes
46.7	1	0	13.7	Now
35.8	0.5	1	5.95	
23.35	0.21	3.76	1.70	Conformal halftime
15.9	0.1	9	0.56	
4.5	0.01	99	0.017	
1.0	0.001	999	0.00044	

Values based on 5-year WMAP data and ΛCDM model computed using WolframAlpha ^®^.

In stark contrast to what is traditionally claimed, the CMB actually tells against a formerly smaller universe and so do the most distant galaxies. The visibility of these has not been reconciled with the idea of a Big Bang. The related attempt to do so has led to a confused use of models that are incompatible with each other. The need for invoking the Expanding View model would disappear if we actually saw mirror images [as in model 2], but in order for galaxies to be seen in this way and the actual isotropy of the CMB to be obtained, the reflector would need to be of all too spectacular stability and flatness - like that required in a telescope of giga-lightyears in length.

## Discussion

Because of the inherent inconsistencies of the standard ΛCDM concordance cosmology, here represented by models 4 and 5, it does not come as a surprise that “ *misconceptions and confusions have long been common in papers on cosmology, also in many by renowned authors*”, as reported by Davis & Lineweaver (2004). These authors deserve credit for having paid attention to those. However, they did not either notice that early events cannot be seen directly. In proceeding without considering reflections (last passage of their section 3.3), they mistook the intersection between our past light cone and the future light cone of the LSS [where a reflection would need to occur] for “ *the points from which the CMB was emitted*” ( Davis & Lineweaver, 2004, p. 101). Although this is not yet beyond the particle horizon of the Big Bang, it would still be off target by half as much as model 5. The confusion arose by equating this particle horizon with the surface of last scattering, which the authors refer to as “ *our effective particle horizon*” ( Davis & Lineweaver, 2004). It also disagrees with the caption of their Figure 1, which presupposes model 5 as such.

When Tolman (1931) considered “ *the highly idealized model of a non-static universe filled with black-body radiation as a thermodynamic fluid*”, he did not discuss the implications of the large size of the universe and the possible absence of a reflective confinement or its equivalent. It deserves to be noted that the time required for cavity radiation to attain a desired degree of homogeneity (after a sufficient number of reflections) increases in proportion to the linear size of the cavity. In a Big Bang universe, this will even with modest demands take much longer than its age. If there is no boundary surface other than one that recedes at *c*, we have seen that any old radiation will eventually disappear from view. In a flat and non-reflective Big Bang universe, this must happen to the radiation from the original LSS, which, thus, cannot remain visible. The CMB must have a different source, whose identification exceeds the scope of this article.

It is futile to consider whether the cosmic inflation theory ( Guth, 1981) might solve the homogeneity problem, because the process this theory postulates is terminated long before recombination. In the present article, the homogeneity at the stage of recombination in a Big Bang universe is not put into question. Instead, it is pointed out that homogeneity will be lost *thereafter*, irrespective of anything that might happen before.

While the irrationality of the assumption about the visibility of radiation from a past epoch in a Big Bang universe, which was disclosed in *The problem*, can be clearly seen in a spacetime diagram such as Figure 1, it may be missed if the ordinary coordinates of time and distance are used, especially if a past light cone is shown (in these coordinates shaped like an avocado seed) that continues below *t* = 1.7 Gyr down to the origin, while it is not made evident that the region it traverses there lies outside the Big Bang universe. For examples see the “avocado seeds” in Davis & Lineweaver (2004), more detailed in Whittle and without any scale under “Manipulating Space-Time Diagrams” in Wright.

The fact that the irrationality has remained unnoticed by professionals is an instance of the ordinary uncritical passing down of human culture, of languages, myths, etc. from generation to generation. In this wider cultural context, science stands out as an exceptional, more critical endeavor that requires practitioners not simply to accept and adopt what they were taught, but to check the relevant assumptions and doctrines for consistency and tenability and to recheck them when premises and/or relevant knowledge change. This may sometimes fail to happen, especially in cases like this, where the presence of an inconsistency became potentially clear only gradually, here after 1965, when a teaching practice had already established itself since Tolman (1934). This practice appears to have prevented the disclosure of the irrationality, which would likely have become obvious after a fresh look at the facts. It is in line with this and with Lakatos’ (1976) analysis of research programs that the rejection of the idea of a Big Bang has been blocked in model 5, although the evidence that requires the rejection has been accepted. Blockage of this kind tends to foster more or less absurd speculation. While scientific journals often tolerate speculative ideas like “inflation” and the “multiverse”, which have been left out of consideration here, it is unfortunate that most of them refuse through prejudice to publish any paper that discredits the “hard core” ( Lakatos, 1976) of the currently accepted doctrine within their field from inside. For editors, it is rational to reject such papers right away: these might threat their reputation if later shown to be erroneous. Also for reviewers who lack a critical attitude against the established practice and doctrine, it is a priori inconceivable that the whole community of well-educated professionals, here mainstream cosmologists, could have made the same cardinal blunder. This holds also in cases like this one, in which the presence of at least one inconsistency is obvious to the uncommitted.

Although the deficiencies disclosed here can be judged as completely unacceptable, other ones need to be addressed as well ( López-Corredoira, 2017; Merritt, 2017; Spergel, 2015; Traunmüller, 2014; Traunmüller, 2018). Just consider that both Λ (dark energy) and CDM (cold dark matter) have remained in the imaginary realm and so merely represent mythical factors or immunizing tactics (also called “conventionalist stratagems”) that protect a doctrine from empirical falsification ( Merritt, 2017). Approaches that rely on such factors are excessively speculative, but inconsistencies such as the two revealed here must be desisted from in any discipline that is meant to qualify as rational. Within standard Big Bang cosmology, there is at least one additional inconsistency that is similarly serious. It is well-known that in this cosmology, any coherent and gravitationally bound objects up to the size of galaxy clusters are exempt from expansion. Only the voids between these clusters are free to expand (Traunmüller, 2018). Under this premise, the matter density within the universe could never have been higher than it uses to be within galaxy clusters – never as high as assumed during the alleged epoch of last scattering. I am not aware of an excuse for this, but suggesting some fancy new physics that might hide inconsistencies is not the preferable way. One should first look for and correct old mistakes that might cause the inconsistencies. One should strive for well-foundedness in the physical principles ( Traunmüller, 2018) rather than merely for a rationalized mythology, but it is, of course, even more fundamental to respect reason at all.

## Data availability

No data are associated with this article.
