# Supplementary material for: Does standard cosmology really predict the cosmic microwave background?
Source: F1000Res. 2021 Sep 23;9:261. Originally published 2020 Apr 16. [Version 6] doi: 10.12688/f1000research.22432.6 (PMC8127010; doi:10.12688/f1000research.22432.6)
Supplement: Peer review history

# Peer review: Reviewer response for version 6

**DOI:** 10.5256/f1000research.77737.r95254

**Published:** 2021-10-04

**Authors:** Marcos C.D. Neves

**Affiliations:** 1 Physics Department, State University of Maringá, Maringá, Brazil

I would like to quote Geoffrey Burbidge to support the conclusion of the paper of Dr. Hartmut Traunmüller (in: Jean-Claude Pecker, Jayant Narlikar, Current Issues in Cosmology. Cambridge University Press, 2006, p. 237-238 ^1^).

 "The contrast between the two perceptions gets further highlighted when one

 notices the large number of speculative concepts that have gone into the standard

 paradigm: The nonbaryonic dark matter, dark energy, phase transitions at energy

 well beyond the range tested in the laboratory, etc. These relate to parts of the

 Universe that will remain forever unobservable and whose physics will remain

 forever untested in the laboratory. However, without making these assumptions the

 theory fails. The fact is that we do not know how galaxies form, and for them to

 form in a big-bang Universe it is necessary to invoke initial density fluctuations and

 a large amount of nonbaryonic matter to make them condense.

 On the other hand, the anomalous evidence ignored by the conventional cosmologists is real, right on our doorstep, and well observable. Surely we need to probe it

 further and in a way that will enable us to understand if any new physics is needed

 here. It is unfortunate that the majority of the cosmology community chooses to

 ignore all of this evidence in the hope that it will go away."

 The BB paradigm is very weak: the epyciles of dark matter and dark energy are weaker than the ptolemaic epicycles. Perhaps, the great error of the nowadays Cosmology is to submit of the assertions of the particle physics, as intended by Steven Weinberg. It is necessary to costruct a new physics based not in epicycles, but in testable hypothesis in our laboratories or in the solar system´s neighborhoods.

Is the topic of the opinion article discussed accurately in the context of the current literature?

Yes

Are arguments sufficiently supported by evidence from the published literature?

Partly

Are all factual statements correct and adequately supported by citations?

Yes

Are the conclusions drawn balanced and justified on the basis of the presented arguments?

Yes

Reviewer Expertise:

history and epistemology of science, cosmology/astronomy, physics and astronomy teaching, art-science relation.

I confirm that I have read this submission and believe that I have an appropriate level of expertise to confirm that it is of an acceptable scientific standard.

## References

[1] : Current Issues in Cosmology. *Cambridge University Press*.2006;237-238

# Peer review: Reviewer response for version 5

**DOI:** 10.5256/f1000research.32621.r81795

**Published:** 2021-05-14

**Authors:** Matthew R. Edwards

**Affiliations:** 1 University of Toronto, Toronto, Canada

This is quite an unusual situation. I was asked to review this paper, even though it has already been reviewed by several competent researchers and sometimes more than once, for different versions. I do not personally subscribe to either the "Big Bang" or "Standard" models, as they rest on very shaky assumptions.  I am sure the other reviewers have studied the mainstream cosmology more than I have and thus I probably cannot add too much to what they have said. The general format here is hard to read, with the long reviewers' reports and multiple versions.  My comments will be very brief.

 As the other reviewers point out, Traunmuller has not accomplished what he set out to do, which is to show an inconsistency in the Standard Model. In my opinion, this is because he has not focused enough on the role of spacetime.  Spacetime is more than space. It is a physical substratum of some kind, one that we don't really understand yet. Think of spacetime as a bundle of optical fibres which can stretch out. During the Big Bang, this bundle expands from a very dense state to a thinner state. The photons and particles are always embedded in these spacetime fibres and can never escape them. The photons can thus never outpace the particles, as Traunmuller supposes. The separation between the particles and between photons, within spacetime, merely increases. So if we were to judge the paper by its own aims, I would have to reject it.

 On the other hand, Traunmuller has done a lot of good work here and I think this could be of general interest, as reflected in the comments of one reviewer who accepted it. Traunmuller's paper is thought provoking at least, and is in many ways far more comprehensible than mainstream cosmology papers. I think it is generally more useful than not. My recommendations are three. I accept the paper with the above noted reservations. The other reviewers' comments are all here to guide readers. I suggest that no further reviews be asked for, as I think it unlikely that a 'better' version can be achieved.  Lastly, it might be best to label this paper as a "Discussion" or "Hypothesis" paper, to alert readers to the fact that we are not expecting a clear result in this paper.

 As a general comment, I would add that cosmology is not in good shape at the moment, and has not been for almost a century.  It is not an area of science where facts can easily be determined. The whole of modern cosmology rests on a few assumptions concerning the Hubble redshift, the CMB, etc.  Many of these assumptions are wrong in my opinion. If I had been asked to review most cosmology papers being published today, I would have had to reject most of them.

Is the topic of the opinion article discussed accurately in the context of the current literature?

Partly

Are arguments sufficiently supported by evidence from the published literature?

Partly

Are all factual statements correct and adequately supported by citations?

Partly

Are the conclusions drawn balanced and justified on the basis of the presented arguments?

Partly

Reviewer Expertise:

I have written a number of papers on gravity and cosmology and edited one book on gravity.

I confirm that I have read this submission and believe that I have an appropriate level of expertise to confirm that it is of an acceptable scientific standard.

# Peer review: Reviewer response for version 5

**DOI:** 10.5256/f1000research.32621.r79938

**Published:** 2021-03-23

**Authors:** Louis Marmet

**Affiliations:** 1 Department of Physics and Astronomy, York University, Toronto, Canada

In his response dated 2021-2-19 the author specifies that he makes the distinction between the "Big Bang" model and the "Standard Model of Cosmology", even if the literature does not usually need to make this distinction. Given this clarification I have read the paper from a different perspective.

   Version 5 of the paper provides a discussion of various Models numbered from 1 through 4, and a fifth *"Expanding View and chronogonic"* model I will refer to as "Model 5". These models are immediately dismissed by the author:

*"Model 1 is clearly incompatible with the assumption that the universe is filled with a homogeneous mixture of matter and blackbody radiation."*  In other words, it is incompatible with the cosmological principle.

   "Model 2" has a problematic "mirror" or "edge", which are just as problematic. It is also incompatible with the cosmological principle.

   "Model 3" has a curvature +1 that is incompatible with observations of the CMB and with galaxy distributions as well.

   "Model 4" is based on "Model 1" and supplemented with an assumption that is contrary to "Model 1": *"that the universe is homogeneously filled with matter and blackbody radiation"*. Since the definition uses an assumption and its contrary, "Model 4" is logically inconsistent.

   The *"Expanding View and chronogonic"* "Model 5" is rejected for the reason that it does not explain the CMB.

   What the author shows in the rest of the paper is that any of the "Models" cannot explain the cosmic microwave background. That is a valid conclusion, but it is rather uninteresting since these "Models" are already rejected for the reasons given on pp. 4 and 5. This reviewer does not understand why five Models are defined, dismissed, and then shown again to be inconsistent.

   However, the author fails to correctly use the terms "Big Bang" model and "Standard Model of Cosmology".

   "Big Bang" models posits no more than the universe is expanding from a hot and dense state, and primordial nucleosynthesis generated the elements we now see. The "Big Bang" model is general and does not say anything about the distribution of matter in the universe. Therefore, neither 'matter is limited to a finite volume' or 'matter is uniform everywhere' contradicts the "Big Bang" model.

   The author is wrong in writing: *"The homogeneity assumption is drastically incompatible with a Big Bang in flat space, in which radiation from past events, such as from last scattering, cannot fail to separate ever more from the material content of the universe."*  The author assumes that the material content of the universe is of limited extent, but the "Big Bang" model does not assume such a thing. Figure 1 shows a possible "Big Bang" model but *not the only* possible "Big Bang" model.

   It is not the "Big Bang" model but "Model 1" that is supplemented with a contradictory assumption by the author. As a result the author incorrectly thinks that this reviewer (and others) *"misinterprets"* what the author says, when in fact it is the author who misinterprets the definition of the "Big Bang" model.

   According to the citation, Tolman considered the *"model of the expanding universe with which we deal ... containing a homogeneous, isotropic mixture of matter and blackbody radiation,"* which clearly means that Tolman assumes there is no limit to the extent of the radiation distribution in space. This is compatible with the "Big Bang" model. The last scattering surface we see today is a two-dimentional spherical cut out of the entire universe at the time of last scattering. In a billion years, we will be receiving light from a larger last scattering surface at a comoving distance of about 48 Gly where matter and radiation was also present.

   The "Standard Model of Cosmology" is based on the "Big Bang" model ( not on "Model 1") and on a possible FLRW solution that fits best the current astronomical observations. The "Standard Model of Cosmology" posits that matter and radiation are distributed uniformly everywhere in the universe. This new supplemented assumption is not contrary to the "Big Bang" model because the latter does not say anything about the distribution of matter. What the author writes: *"... filled with a photon gas within an imaginary box whose volume V"* is incorrect since the photon gas is not limited to a finite volume at the time of last scattering.

   None of the five "Models" corresponds to the "Standard Model of Cosmology", so the fact that they are falsified has no bearing on whether or not the "Standard Model of Cosmology" can predict the cosmic microwave background.

 A comment on the author's response: *"... a Big Bang model is described, and the imaginary box does not exist in nature. Despite this, the calculations are done as if it was present. Ryden here merely follows a tradition, but this is the cardinal blunder I mention in the second passage under Model 2. Since there is actually no such box..."*  Indeed, this is another blunder of "Model 2" defined by the author. However, there is no need for such a box in the "Standard Model of Cosmology" since, unlike in "Model 2", matter and radiation fill the expanding universe entirely.

 Answers to the questions:

- *Is the topic of the opinion article discussed accurately in the context of the current literature?*

    No, the author incorrectly equates the "Standard Model of Cosmology" with some of his "Models".

- *Are all factual statements correct and adequately supported by citations?*

    No, e.g. the papers cited (paragraph starting with "Model 1") do presuppose that radiation and matter entirely fill the expanding universe, contrary to the description of "Model 1" which assumes a limited volume containing matter.

- *Are arguments sufficiently supported by evidence from the published literature?*

    No, the five Models defined in the article do not correspond to anything found in the literature.

- *Are the conclusions drawn balanced and justified on the basis of the presented arguments?*

    No, the paper shows that the five "Models" are falsified, but incorrectly concludes that a different model, the "Standard Model of Cosmology", is inconsistent.

Is the topic of the opinion article discussed accurately in the context of the current literature?

No

Are arguments sufficiently supported by evidence from the published literature?

No

Are all factual statements correct and adequately supported by citations?

No

Are the conclusions drawn balanced and justified on the basis of the presented arguments?

No

Reviewer Expertise:

Precision spectroscopy, time standards, GPS systems, relativity, observational astronomy.

I confirm that I have read this submission and believe that I have an appropriate level of expertise to state that I do not consider it to be of an acceptable scientific standard, for reasons outlined above.

# Response

**Published:** 2021-09-15

**Authors:** Hartmut Traunmüller

**Affiliations:** Stockholm University, Sweden

**Reviewer Louis Marmet’s comment**: The author specifies that he makes the distinction between the “Big Bang” model and the “Standard Model of Cosmology”, even if the literature does not usually need to make this distinction. Given this clarification, I have read the paper from a different perspective.

    Version 5 of the paper provides a discussion of various Models numbered from 1 through 4, and a fifth “ *Expanding View and chronogonic” *model I shall refer to as “Model 5”. These models are immediately dismissed by the author:

    “ *Model 1 is clearly incompatible with the assumption that the universe is filled with a homogeneous mixture of matter and blackbody radiation.” *In other words, it is incompatible with the cosmological principle.

    “Model 2” has a problematic “mirror” or “edge”, which are just as problematic. It is also incompatible with the cosmological principle.

    “Model 3” has a curvature +1 that is incompatible with observations of the CMB and with galaxy distributions as well.

    “Model 4” is based on “Model 1” and supplemented with an assumption that is contrary to “Model 1”: “ *that the universe is homogeneously filled with matter and blackbody radiation”*. Since the definition uses an assumption and its contrary, “Model 4” is logically inconsistent.

    The “ *Expanding View and chronogonic” “*Model 5” is rejected for the reason that it does not explain the CMB.

**Author’s response: **In the modified final version, I distinguish a relic radiation model from a chronogonic expanding view model. This agrees with the Reviewer’s distinction between model 4 and 5. Model 4 is a Big Bang model that is marred by a blunder, while Big Bang cosmogony is dismissed in model 5, in which the universe is infinite to begin with.

**Reviewer’s comment**: What the author shows in the rest of the paper is that any of the “Models” cannot explain the cosmic microwave background. That is a valid conclusion, but it is rather uninteresting since these “Models” are already rejected for the reasons given on pp. 4 and 5. This reviewer does not understand why five Models are defined, dismissed, and then shown again to be inconsistent.

**Author’s response:** My popularization, *Embarrassing blunders in big bang cosmology*, is more pedagogically structured.

**Reviewer’s comment: **However, the author fails to correctly use the terms “Big Bang” model and “Standard Model of Cosmology”.

**Author’s response:** I adopt the common use of terms (as in, e.g., https://phys.org/news/2015-12-big-theory.html), according to which “Big Bang models” are GR-based cosmological models in which the universe expands persistently from a hot and dense “primeval fireball” (Peebles’ favorite term) or “primordial fireball”. Thus, they comprise a finite, expanding region filled with matter and radiation. In standard cosmology, a Big Bang is assumed for some aspects while it is ignored for others, as when a radiation source is claimed to be more distant than 23.4 comoving Gly. Before judging correctness, one has to choose one of the models and reject the other. I show that, in a Big Bang universe, we cannot see the primeval fireball. If one, instead, assumes the universe to have been infinite at the onset of time, as some like the reviewers Indranil Banik and Louis Marmet do, one has either already rejected the idea of a Big Bang or confused it with the very different idea of an Expanding View.

**Reviewer’s comment**: …“The “Big Bang” model is general and does not say anything about the distribution of matter in the universe. Therefore, neither ‘matter is limited to a finite volume’ or ‘matter is uniform everywhere’ contradicts the “Big Bang” model.

**Author’s response**: Big Bang models are obtained from GR by presupposing that the modeled universe remains homogeneously filled with a fluid of matter and radiation. I point out that a Big Bang universe does not allow such a state to be maintained. The denied contradiction is absent because in Big Bang models the everywhere is limited to a finite volume.

**Reviewer’s comment**: The author is wrong in writing: “ *The homogeneity assumption is drastically incompatible with a Big Bang in flat space, in which radiation from past events, such as from last scattering, cannot fail to separate ever more from the material content of the universe.” *The author assumes that the material content of the universe is of limited extent, but the “Big Bang” model does not assume such a thing. Figure 1 shows a possible “Big Bang” model but *not the only* possible “Big Bang” model.

**Author’s response:** My statement holds for what I (and most others) mean with the “Big Bang”, in which everything can be traced back to a compact primeval fireball. The Reviewer appears, instead, to prescribe an Expanding View model, in which the spatial extension of the universe was never limited while more of it came gradually into view. However, in mainstream tradition, the homogeneity of the CMB is maintained not by widening the universe like this (model 5), but by narrowing it to a region with the comoving diameter of the last scattering surface (model 4). This is the relic radiation blunder.

**Reviewer’s comment**: It is not the “Big Bang” model but “Model 1” that is supplemented with a contradictory assumption by the author. As a result the author incorrectly thinks that this reviewer (and others) “ *misinterprets” *what the author says, when in fact it is the author who misinterprets the definition of the “Big Bang” model.

**Author’s response**: My “model 1” represents a Big Bang model that is neither marred by the relic radiation blunder nor confused with an Expanding View model.

**Reviewer’s comment**: According to the citation, Tolman considered the “ *model of the expanding universe with which we deal ... containing a homogeneous, isotropic mixture of matter and blackbody radiation,” *which clearly means that Tolman assumes there is no limit to the extent of the radiation distribution in space. This is compatible with the “Big Bang” model.

**Author’s response**: The citation is actually taken from Alpher and Herman (1975). It reads like a warning: do not take our conclusions as valid if the universe is not like this. In believing that it is, the authors appear to have followed Tolman (1934), who had begun his studies of the thermal properties of the universe before he had become familiar with GR based models. He thought erroneously that his earlier conclusions would still hold also in these, and none of his followers corrected this.

**Reviewer’s comment: **The last scattering surface we see today is a two-dimensional spherical cut out of the entire universe at the time of last scattering. In a billion years, we will be receiving light from a larger last scattering surface at a comoving distance of about 48 Gly where matter and radiation was also present.

**Author’s response:** The “last scattering surface” is just a theoretical construct within a cosmogonic Big Bang model, and I think I made it clear that such a model does not allow us to see this surface. We see something else.

**Reviewer’s comment: **The “Standard Model of Cosmology” is based on the “Big Bang” model ( not on “Model 1") and on a possible FLRW solution that fits best the current astronomical observations. The “Standard Model of Cosmology” posits that matter and radiation are distributed uniformly everywhere in the universe. This new supplemented assumption is not contrary to the “Big Bang” model because the latter does not say anything about the distribution of matter.

**Author’s response**: FLRW models are obtained from GR by assuming that matter and radiation are distributed uniformly in the space that they describe. This is not only posited in the alleged “Standard Model of Cosmology”. What is new there is, instead, the ab initio presence of an infinite universe, which contradicts the model of a finite expanding universe that is used for the explanation of other aspects.

 Reviewer’s continued comment: What the author writes: “ *... filled with a photon gas within an imaginary box whose volume V” *is incorrect since the photon gas is not limited to a finite volume at the time of last scattering.

**Author’s response:** I consider Ryden´s textbook as representative of the present standard approach to cosmology (checked for orthodoxy by several authorities in the field), and it says: “Consider a region of volume *V* which expands at the same rate as the universe, so that *V* prop. *a*( *t*) ^3^. The blackbody radiation in the volume can be thought as a photon gas with energy density ε _γ_ = *αT* ^4^ *.*” This is model 4 - neither model 1 nor model 5.

**Reviewer’s comment**: A comment on the author’s response: “ *... a Big Bang model is described, and the imaginary box does not exist in nature. Despite this, the calculations are done as if it was present. Ryden here merely follows a tradition, but this is the cardinal blunder I mention in the second passage under Model 2. Since there is actually no such box...” *Indeed, this is another blunder of “Model 2” defined by the author. However, there is no need for such a box in the “Standard Model of Cosmology” since, unlike in “Model 2”, matter and radiation fill the expanding universe entirely.

**Author’s response:** One can avoid the relic radiation blunder by following Tolman’s reasoning. This is clearly possible in universes with zero curvature if these were large enough at the onset of time. However, this condition implies already a rejection of the idea of a cosmogonic Big Bang.

**Reviewer’s comment**: None of the five “Models” corresponds to the “Standard Model of Cosmology”, so the fact that they are falsified has no bearing on whether or not the “Standard Model of Cosmology” can predict the cosmic microwave background.

**Author’s response**: Strictly speaking (I did not do so and allowed the common usage), there is no “standard model of cosmology” at all. Instead, there is a standard approach that involves three contradictory models, which are used for separate aspects.

    The first one is the prototypical Big Bang model (model 1). This model suggests a cosmic redshift and a last scattering surface. However, it predicts the radiation from the latter to be invisible by now. In this model, the universe has a constant finite mass and it must expand at *c* in order not to hinder radiation.

    The second one (model 4) is a Big Bang model that is marred by the relic radiation blunder. It fills, at any given cosmic time after last scattering, a volume that is smaller than that in model 1 (but equal to that in model 2). This is how the CMB properties are modeled, such as the evolution of its temperature as *T* ～ 1/ *a*( *t*) (eq. 6.3 in Peebles, 1993) from 3000 K to 2.7 K.

    The third one (model 5) is an Expanding View model, which uses to be introduced tacitly and fills a volume that is larger than that in model 1. It appears to be the result of using distance measures in whose calculation the spatial limitation of the universe given by the Big Bang model had been and still is ignored by mistake. Then only the temporal limitation remains. Accepting these standard distance measures (or Tolman’s mentioned approach) is equivalent to rejecting the idea of a cosmogonic Big Bang. It may be that similar distance measures are actually valid in a tenable cosmology (no big bang), but in this case the CMB and its homogeneity must have a different origin.

# Peer review: Reviewer response for version 4

**DOI:** 10.5256/f1000research.29803.r74224

**Published:** 2020-11-30

**Authors:** Louis Marmet

**Affiliations:** 1 Department of Physics and Astronomy, York University, Toronto, Canada

Summary

 The paper "Does standard cosmology really predict the cosmic microwave background?" by Hartmut Traunmüller examines the claim made by the Big Bang model that the cosmic microwave background (CMB) has its origin in the thermal light of last scattering at the time of recombination. The paper explains that the CMB originated from matter that was contained inside the last scattering surface (LSS) with comoving radius of about 0.95 Gly. It is argued that for light to reach our position from all directions, it would take at most 1.6 My (cosmic time). Since the universe is much older than that, radiation from the LSS has past us a long time ago, and therefore the CMB observed today cannot originate from the LSS.

 Three models other than the Big Bang are first examined in an attempt to explain the CMB: a flat universe without boundary, a flat universe with a reflecting "edge", and a positively curved universe where light can return to its initial position. None of those can provide a satisfactory explanation for the CMB. The present standard model of cosmology (labelled model 4) is then examined in more details, but the conclusion is still that "the CMB actually tells against a formerly smaller universe."

 The author concludes that cosmologists have not noticed that early events cannot be seen directly, that ΛCDM is irrational, and that publications that discredit the "hard core" doctrine are rejected by scientific journal.

Review

 Big Bang cosmology has many known serious problems as is frequently reported in papers about the need for new physics, tensions in the evaluation of cosmological parameters, and a crisis in cosmology. However, the present paper is not exempt from the misconceptions and confusions that are common in papers on cosmology.

 The author assumes that at the time of recombination, there was only matter inside a volume with a 0.95 Gly comoving radius, so that light released after recombination is only emitted from within this volume. This is incorrect. Despite having read Ryden's 'Introduction to Cosmology' the author ignores that matter is assumed to exist beyond the horizon:

 'The stars *beyond some finite distance, called the horizon distance*, are invisible to us because their light hasn't had time to reach us yet' (Ryden 'Introduction to Cosmology' 2016, p. 10) ^1^.

 As a result of this misconception, the author incorrectly plots a short "red horizontal dash" in Fig. 1. But why would matter only be created within a given comoving radius from us? The correct depiction would show the red line extending beyond a comoving distance of 46 Gly. Big Bang cosmology claims that there are stars beyond the horizon distance, and since the LSS is always inside the expanding horizon (Ryden, Fig. 9.3 ^1^), light emitted at the time of recombination can always be seen.

 The author continues: "The idea that the CMB comes directly, although redshifted, from a LSS emerged only after 1965. It is not clear how the early followers of Tolman (1934) thought about this, but it requires normally a confinement in order to keep blackbody radiation within a region" (p. 3, right)

 This shows that the author considers the 'last scattering surface' as contained within a limited "region" of comoving space. This is *not* what is claimed by the Big Bang model.

 The author makes the following argument (p. 3-4):

 "If the CMB originated at the LSS and all matter originated within the region enclosed by this surface..." - Again, this second statement is in error because there was matter outside the LSS. - "...then, how can it be that we can see the light?" - Because it comes from matter that was at a comoving distance beyond 46 Gly away.

 Unfortunately, this incorrect interpretation of the Big Bang model early in the paper voids the rest of the discussion.

 Near the end of the paper, the author touches on arguments that were eventually used to produce inflation theory without citations ("black-body radiation...time required for cavity radiation to attain a desired degree of homogeneity...this will even with modest demands take much longer than its age"). These are valid arguments that have been studied earlier. While inflation theory has problems of it's own, one cannot argue against these models based on straw man arguments.

 Based on all these observations, I do not recommend this article being indexed.

 A previous reviewer (I. Banik) correctly identified the problem with the author's argument. The reviewer's comment "...what would be more useful is to draw two more multiple orange last scattering surface (LSS) cones translated along x,..." precisely matches my understanding of the Big Bang model. For example in Ryden's book, Fig. 9.3 ^1^ proves that the standard model considers that there is matter beyond the LSS. This is even what the author tries to describe in the paragraph starting with "In a model that is slightly less obviously untenable..." but the idea is abandoned.

 Concerning the specific questions, the answers are 'no' to every one of them.

*Is the topic of the opinion article discussed accurately in the context of the current literature?*

- No, the author misunderstood the present standard model.

*Are all factual statements correct and adequately supported by citations?*

- No, some are based on a misunderstanding of the standard model.

*Are arguments sufficiently supported by evidence from the published literature?*

- No, the present literature does not explain the Big Bang model the way the author has understood it.

*Are the conclusions drawn balanced and justified on the basis of the presented arguments?*

- No, they can't be because of the author has an incorrect conception of the standard model of cosmology.

Is the topic of the opinion article discussed accurately in the context of the current literature?

No

Are arguments sufficiently supported by evidence from the published literature?

No

Are all factual statements correct and adequately supported by citations?

No

Are the conclusions drawn balanced and justified on the basis of the presented arguments?

No

Reviewer Expertise:

Precision spectroscopy, time standards, GPS systems, relativity, observational astronomy.

I confirm that I have read this submission and believe that I have an appropriate level of expertise to state that I do not consider it to be of an acceptable scientific standard, for reasons outlined above.

## References

[1] : Introduction to Cosmology.2016;

# Response

**Published:** 2020-12-18

**Authors:** Hartmut Traunmüller

**Affiliations:** Stockholm University, Sweden

Reviewer’s comment: The author assumes that at the time of recombination, there was only matter inside a volume with a 0.95 Gly comoving radius, so that light released after recombination is only emitted from within this volume. This is incorrect. 

 Author’s response: I assume this of a proper Big Bang universe, because ≈ 0.95 Gly is the comoving radius such a universe is calculated to have at the time of recombination, and there was *nothing* outside it at that time (not even a physical vacuum). In a new second passage under *Model 4*, it is now clarified how the standard model deviates from this.

 Reviewer’s comment: Despite having read Ryden's 'Introduction to Cosmology' the author ignores that matter is assumed to exist beyond the horizon: 'The stars *beyond some finite distance, called the horizon distance*, are invisible to us because their light hasn't had time to reach us yet' (Ryden 'Introduction to Cosmology' 2016, p. 10) ^1^.

 Author’s response: This quotation is taken from a passage (end of section 2.1) in which Olbers’ paradox is discussed. It refers to our observations in this context and not to what a Big Bang model predicts. The statement is, instead, in line with an Expanding View model.

 Reviewer’s comment: As a result of this misconception, the author incorrectly plots a short “red horizontal dash” in Fig. 1. But why would matter only be created within a given comoving radius from us? (Author: not only from our galaxy, but from any other galaxy as well!)

 Author’s response: The answer is obvious enough: because the Big Bang universe was no lager then – no wider than the short red horizontal dash.

 The reviewer consistently misinterprets what I say about a proper flat Big Bang model as incorrect statements about the standard model, in which such a model is supplemented with a *contradictory* model. I did point out the conflict between the constituent models in the first two passages under *Model 4*. The new second passage inserted there is intended to prevent the misinterpretation. It is only the contradictory supplement that allows anything to exist outside the proper Big Bang universe, i.e., below the golden V-shaped band in my Figure 1.

 As I explain in the first passages under *Model 4*, in standard cosmology, which is taught by Ryden, an “Expanding Universe” (Big Bang) model is supplemented with a contradictory “Expanding View” model.  I have now made it explicit that I had to coin the attributes “Expanding View” and “chronogonic” myself (inspired by correspondence with Barbara Ryden). In the literature, the supplementary model uses to be invoked informally, without being named. Together with the common but misleading practice of referring to the entire standard model as a Big Bang model, this dampens the awareness about the presence and contradictoriness of the supplement, as demonstrated by the comments from reviewers I. Banik and L. Marmet.

 The calculations of densities, redshifts, temperatures, etc. are still done in a Big Bang model whose comoving radius was ≈ 0.95 Gly when it became transparent. Consider what Ryden says in section 2.5 of her book: “The blackbody radiation that fills the universe today can be explained as a relic of the time when the universe was sufficiently hot and dense to be opaque. However, at the time the universe became transparent, its temperature was 2970 K. The temperature of the CMB today is 2.7255 K, a factor of 1090 lower. The drop in temperature of the blackbody radiation is a direct consequence of the expansion of the universe. Consider a region of volume *V *that expands at the same rate as the universe, so that *V *∝ a( *t*) ^3^. The blackbody radiation in the volume can be thought of as a photon gas with energy density *ε _γ_* = *αT* ^4^. ... The photon gas within our imaginary box follows the laws of thermodynamics; ...” Some of this and a reference to the corresponding section in the book is now inserted.

 Here, a Big Bang model is described, and the imaginary box does not exist in nature. Despite this, the calculations are done as if it was present. Ryden here merely follows a tradition, but this is the cardinal blunder I mention in the second passage under *Model 2*. Since there is actually no such box, there is no way for any relic radiation to remain within the volume *V *∝ a( *t*) ^3^ that is represented by the LSS-wide region between the two dashed vertical lines in my Figure 1. The  radiation cannot fail to escape from this region at *c* (within the golden V). Since this is missed in Ryden’s description, the model is flawed, but before being supplemented, it is still a Big Bang model in which nothing at all exists below the golden V. (Approximately this is now said under *Model 4* and contrasted with Tolmans approach that defies the Big Bang model already at this point.)

 Reviewer’s comment: Near the end of the paper, the author touches on arguments that were eventually used to produce inflation theory without citations (“black-body radiation...time required for cavity radiation to attain a desired degree of homogeneity...this will even with modest demands take much longer than its age”). These are valid arguments that have been studied earlier. While inflation theory has problems of it's own, one cannot argue against these models based on straw man arguments.

 Author’s response: It is futile to consider whether the cosmic inflation theory (Guth, 1981) might solve the homogeneity problem, because the process this theory postulates is terminated long before recombination. In the present article, the homogeneity at the stage of recombination in a Big Bang universe is not put into question. Instead, it is pointed out that homogeneity will be lost *thereafter*, irrespective of anything that might happen before. (New 3 ^rd^ passage under *Discussion*.)

 Reviewer’s comment: A previous reviewer (I. Banik) correctly identified the problem with the author's argument. The reviewer's comment “...what would be more useful is to draw two more multiple orange last scattering surface (LSS) cones translated along x,...” precisely matches my understanding of the Big Bang model. For example in Ryden's book, Fig. 9.3 ^1^ proves that the standard model considers that there is matter beyond the LSS. This is even what the author tries to describe in the paragraph starting with “In a model that is slightly less obviously untenable...” but the idea is abandoned.

 Author’s response: Fig. 9.3 in Ryden’s book (Fig. 8.4 in the 2017 edition) does not depict the Big Bang model. It depicts the supplementary Expanding View model, which is typically introduced by a figure like this. This supplementary model is meant to bring our actual observations into agreement with the theoretical expectations of a Big Bang model. This is attempted by turning the Big Bang universe inside out. This results in the ring-shaped LSS shown in Ryden’s Fig. 8.4, which corresponds to the two peripheral short red horizontal dashes in my Figure 1. Their location is spatially most remote from the LSS in the unsupplemented Big Bang model (the red horizontal dash close to the origin) by which the properties of the CMB are still derived. Standard cosmology operates, thus, with two drastically different locations of the same last scattering event, and this is irrational. (Approximately this is now said)

 By the way, simply turning the Big Bang model inside out does not invalidate the initial statements under “ *The problem*”. Even if this is done, which is a drastic error, it still needs to be considered that light propagates from the LSS faster than the constituent matter of an observer can have moved. This precludes a common place of origin for matter and the CMB  also at the periphery of the visible universe. (Now made explicit.)

 It is not either possible to replace the Big Bang model by the Expanding View model, because the latter does not predict the properties of the CMB based on its own premises – not even its very existence and that of a cosmic redshift.

# Peer review: Reviewer response for version 3

**DOI:** 10.5256/f1000research.27713.r68925

**Published:** 2020-09-18

**Authors:** Abhas Mitra

**Affiliations:** 1 Homi Bhabha National Institute, Bhabha Atomic Research Centre, Mumbai, Maharashtra, India

***Updated on 23/09/2020 to incorporate the references below which the reviewer wanted to be added.***

 I appreciate the fact that the author has the courage to raise some basic questions about the Big Bang Model (BBM) and its modern version (LCDM). In particular,

 (i) He has argued that we cannot see primordial radiation supposed to be emanating from the Last Scattering Surface (LSS) if the universe is indeed flat and without a “reflective surface”. On the other hand, he argues that the LSS should be visible only for a closed Universe.

 (ii) He argues that this must be so because while radiation streams with speed c, the matter moves with subluminal speed and a flat universe has no “reflective surface” (boundary).

 (iii) He has also raised a point that “traditional calculation of the CMB temperature is flawed”.

 Although I think that BBM  and in particular the “standard” LCDM cosmology suffer from several inconsistencies, both from theoretical and observational perspectives (Pecker  1997, López-Corredoira 2017), the points (i) & (ii) raised by the author are results of confusion and misunderstanding.

 1. First, visibility of LSS has got nothing to do with the issue: “ light escapes faster than matter can move, (and we) ….are made of matter from this very source”

 When we observe any object say a table in the room, our eyes or our telescopes need to receive the light emanating from the surface of the table, and we do not require the matter constituting the table to strike our eyes or our telescopes. Thus it is immaterial whether matter of the table moves towards us with the speed of light or subluminal speed or with zero speed.

 2. Both the Model 1 & 2 mentioned by the author are flat models (k=0). However he mentions of  “reflective boundary” in one case. There is no such different versions of the flat model, and the idea of a “reflective boundary” is incorrect and I am afraid shows basic misconceptions. In fact, much of the prolonged discussions are hardly comprehensible and I am afraid, results from confused thinking and misunderstanding. There just cannot be any boundary of the universe.

 2. If there is a homogeneous plasma, under ideal conditions, it will remain so during expansion or contraction irrespective of the strength of coupling between ionized matter and photons. However, the photons will get constantly scattered and remain tightly tied up with the plasma as long as the Thompson scattering cross section will be sufficiently larger than unity. During such a stage, a section of the plasma will not be able to see distant sections because photons from distant sections are not able to free stream.

 3. During expansion, at some stage, the density of the plasma will decrease so much that the plasma – photon scattering cross-section will drop below unity, and photons will be able to free stream. Suppose this happens when the radius of the plasma is R(t _0_). Then for  t> t _0_, any section of the plasma, which may have condensed to neutral matter, lying beyond R(t) > R(t _0_), will be able to see the region beyond R(t _0_). And it would appear that all the light his receiving is emanating from the LSS: R(t _0_).

 4. Thus the LSS is sometimes called the cosmic photosphere, by analogy with the visible `surface' of the Sun where radiation produced by nuclear reactions is last scattered by the solar material. And contrary to what the author insists, visibility of the LSS has nothing to with whether universe is closed, where there could be return path of photons, or open/flat, where there cannot be any return path of photons.

 5. Having mentioned my above critique, let me tell that, yet there are reasons for introspection of some of the points raised by the author.

 The observed spectral shift of the radiation from the cosmological sources is most elegantly explained by the HYPOTHESIS of an “Expanding Universe”.

 However this idea is fraught with many conceptual difficulties (Chodorowski 2005, 2007a, 2007b; Peacock 2008) and which in turn lead to several theoretical confusions (Baryshev 2008, 2015).

 But even if we ignore such puzzles and paradoxes, we need to ponder whether the idea of an expanding universe makes sense for flat (k=0) and open (k= -1) FRW models.

 It is only the closed (k= +1) FRW model, that the PROPER volume

 V ~ a(t) ^3^

 And which can increase from V=0 to a V=Finite state. The popular analogy of an expanding universe with an inflating balloon actually corresponds to this closed model.

 But for both flat (k=0) and open models (k=-1)  one always has (Tolman  1962):

 V=Infinite

 In order to appreciate this fact, think of a flat sheet of paper for a 2-D “open” model. Since the universe must NOT have any edge, the flat 2-D space must be infinite. And same is true for an open model.

 Thus though mathematically, one may talk about expanding flat and open FRW models in terms of apparently changing scale factor a(t), the hypothesis of an “expanding universe” may be meaningful only the closed model having finite proper volume.

 6. All concepts of Thermodynamic were developed for systems which are finite. And one wonders whether such concepts are extendable for systems having INFINITE proper volumes. And then the question arises whether the concept of “thermal equilibrium” of an infinite system is definable at all.

 7. Even if we admit that the idea of thermal equilibrium is valid even for infinite flat and open FRW models, let us recall that the original Big Bang model of Lemaitre was a COLD model, with no attendant idea of any “thermal equilibrium” or “temperature” (T) of the radiation.

 The idea of HOT Big Bang model was due to Gammow, and it turns out that for an assumed radiation dominated universe, temperature

 T(t) ~1/a(t)

 One however cannot determine T(t) from any first principle since the constant of proportionality in the foregoing equation is not known.

 One may still assign value of the T(t) with additional assumptions about the desired model, and which in a sense is some sort of tautology. That is the reason that the estimates of T for CMB initially varied from author to author.

  8.  We know that, the key to the origin of kinematical pressure and temperature of a fluid is the mutual collisions among its constituent particles. On the other hand, collision is absent only for a mathematical fluid termed as “DUST” which has no pressure, no temperature.

 In the ideal BBC, all test particles (galaxies in present era) are receding away from one another without any mutual collision. Thus in the ideal BBC, which involves assumptions of perfect homogeneity and isotropy, the fluid is a dust. This has been shown specifically by expressing g _00_ in terms of pressure and density (Mitra 2011a,b, 2012). The fact that g _00_=1 for the ideal BBM, leads to p=0. Then in the absence of any collision, temperature of the BBM fluid T=0 too. Thus ideal BBM should be COLD and not HOT.

  9. Energy is conserved for a system which has a timelike Killing vector. And by noting that FRW metric has no such Killing vector, one may declare that total energy of the FRW universe need not be conserved. However, any system can gain or lose energy only by interacting with the REST OF THE UNIVERSE. But for the UNIVERSE, there is no REST OF THE UNIVERSE, and thus its total energy (matter plus gravitation) must be conserved.

 Following Einstein’s definition of  Lagrangian density and gravitational field energy density (pseudo tensor), Tolman  (1930, 1962) derived a general formula for the total matter plus gravitational field energy ( *P _0_*) of an arbitrary self-gravitating system (Landau & Lifshitz 1962). And  by using the Tolman ansatz, in a detail study, I worked out an expression for *P _0_* for the FRW universe (Mitra 2010). It was found that

 P_0 = P _0_ (t)

  This study led to 3 important results:

  

1. In order that matter plus gravitation energy momentum density is position independent, only the k=0 flat model should be admissible.
2. In order that a free falling observer notices no gravitational field in his local Lorentz Frame, Cosmological Constant should be zero: Lambda =0.
3. If energy momentum conservation would be violated, there could be eruption of unlimited energy anywhere anytime out of nothingness. And if energy conservation has to be honored, FRW model must be STATIC with no contraction or expansion.

The foregoing condition may also be obtained in the following way. Total energy of FRW universe can be also obtained by super potentials associated with the same Einstein pseudo tensor; and it is found that (Rosen 1994, Xulu 2003):

 P _0_ =0

 The equality between the two equivalent expressions of  P(t) =P _0_ =0  is possible only in two cases (Mitra 2010):

  

1. FRW universe is actually static
2. If mathematically, one would concept a dynamic FRW model, then, one should tacitly have a VACUUM model with rho =0, Lambda =0.
3. In other words, real physical universe having inhomogeneous distribution of lumpy matter cannot be described by BBM. 

10. In curved spacetime, the 1 ^st^ Law of Thermodynamic must involve proper volume element (dV)  rather than the coordinate  volume element. Accordingly, I formulated this law for an adiabatically evolving fluid which obeys particle number conservation (Mitra 2011c):

*dE *+ 4 *π p R*2 ^2^  sqrt{-g _00_ g _rr_) *(overdot R )dt * = 0

 where (overdot R) is the temporal rate of change of the area coordinate R. For an isotropic and homogeneous universe no section of the fluid can do any work on another section, and the net energy of each section E must be conserved. This seems to be possible only when

  

1. Pressure of the fluid p=0 (as obtained earlier), or
2. Universe is static: (overdot R) =0

 

 11. In fact, from 4 additional independent directions, it has been shown that adhoc cosmological constant should indeed be zero (Mitra 2011d, 2012b, 2013a,b). Let me discuss only one of the 4 results. In it known that the vacuum de-Sitter model can be expressed both as a dynamical FRW model (in comoving coordinates) and also a STATIC model in curvature coordinates (Mitra 2012b, 2015). By using the dynamic FRW model, one finds that the

 Expansion Scalar = sqrt(3 L)

 where L is the Cosmological Constant. However, from physical perspective, for the original STATIC de-Sitter model,

 Expansion Scalar =0

 Since we are talking about a SCALAR, this contraction cannot be explained away as any “coordinate effect”. On the other hand, such a contradiction can be resolved by realizing that the adhoc parameter

 L =0.

 Since the most likely candidate for Dark Energy of the LCDM model is none other than the Cosmological Constant, 4 independent proofs that L=0 strongly suggests that the so-called “Dark Energy” is an illusion arising from the attempt to explain complex lumpy universe by an oversimplified model which requires perfect homogeneity and isotropy (Mitra 2013b, 2014a).

  11. NON-BARYONIC DARK MATTER?

 While there are indeed evidences for existence of unseen gravitating matter at galactic scales, there is no evidence that 

1. Such a Dark Matter is non-baryonic
2. And present as a background matter permeating entire universe, even inter galactic spaces.

 In the BBM, one key parameter is the baryon to photon ratio (eta) and the deuterium abundance  in BB Nucleosynthesis  is highly sensitive to the value of eta.  The observed abundance of (D /H) ~ 2.8x 10 ^(-5)^ demands eta ~ 5. 10 ^-(10)^.  And this in turn leads to a  paltry value of Omega _b_  ~0.04, where Omega _b_ is the ratio of baryon mass density to the critical density needed for a closed (k=+1) universe. And this is the reason that BBM requires most of the matter to be non-baryonic Dark Matter.

 However, despite frantic search for 30 years, there is no evidence for such a non-baryonic DM. Even if a proposed exotic DM matter particle, such as “axion” would be detected, that would be no confirmation that 70% of the unseen matter is made of axions.

 12. Hypothesis of Inflation: BBM requires an early inflationary phase when the size of the universe shot up exponentially. Though, in principle a(t) may have any mathematical form it appears that BBM has *tacit and latent* self-consistency constraints (Mitra 2014b):

 (i) It is spatially flat k=0

 (ii) And its scale factor a(t)∝t

 If this is true, the inflationary phase badly required by BBM is not admissible.

 Thus both classic BBM and its new version LCDM model suffers from fundamental discrepancies and problems. To this extent, the author is correct. But the way he has attempted to make such a strong statement, I am afraid, is not valid.

Is the topic of the opinion article discussed accurately in the context of the current literature?

No

Are arguments sufficiently supported by evidence from the published literature?

No

Are all factual statements correct and adequately supported by citations?

No

Are the conclusions drawn balanced and justified on the basis of the presented arguments?

No

Reviewer Expertise:

General Relativity, Cosmology, Astrophysics

I confirm that I have read this submission and believe that I have an appropriate level of expertise to state that I do not consider it to be of an acceptable scientific standard, for reasons outlined above.

## References

[1] : Expanding Space: The Root of Conceptual Problems of the Cosmological Physics. *Cornell University*.2008;1:20-30Reference source

[2] : Paradoxes of the Cosmological Physics in the Beginning of the 21-st Century, Particle and Astroparticle Physics, Gravitation and Cosmology: Predictions, Observations and New Projects. *World Scientific Publishing*.297-307Reference source

[3] : Cosmology under Milne's shadow. *Publ. Astron. Soc. Austral*.2005; Reference source

[4] : Is Space Really Expanding? A Counterexample. *Old and New Concepts of Physics*.2007;4(1) : 10.2478/v10005-007-0002-215-3310.2478/v10005-007-0002-2

[5] : A direct consequence of the Expansion of Space?. *Monthly Notices of the Royal Astronomical Society*.2007;378(1) : 10.1111/j.1365-2966.2007.11766.x239-24410.1111/j.1365-2966.2007.11766.x

[6] : L. D. Landau and E. M. Lifshitz, The Classical Theory of Fields. Revised second edition. Translated from the Russian. IX + 404 S. m. 19 Abb. Oxford/London/Paris/Frankfurt 1962. Pergamon Press. Preis geb. 80s. net. *ZAMM - Zeitschrift für Angewandte Mathematik und Mechanik*.1963;43(6) : 10.1002/zamm.1963043061110.1002/zamm.19630430611

[7] : Tests and Problems of the Standard Model in Cosmology. *Foundations of Physics*.2017;47(6) : 10.1007/s10701-017-0073-8711-76810.1007/s10701-017-0073-8

[8] : Einstein energy associated with the Friedmann–Robertson–Walker metric. *General Relativity and Gravitation*.2010;42(3) : 10.1007/s10714-009-0863-1443-46910.1007/s10714-009-0863-1

[9] : The matter in the Big-Bang model is dust and not any arbitrary perfect fluid!. *Astrophysics and Space Science*.2011;333(1) : 10.1007/s10509-011-0635-8351-35610.1007/s10509-011-0635-8

[10] : Why the Big Bang Model Cannot Describe the Observed Universe Having Pressure and Radiation. *Journal of Modern Physics*.2011;02(12) : 10.4236/jmp.2011.2121771436-144210.4236/jmp.2011.212177

[11] : Macroscopic Form of the First Law of Thermodynamics for an Adibatically Evolving Non-singular Self-gravitating Fluid. *Foundations of Physics*.2011;41(9) : 10.1007/s10701-011-9557-01454-146110.1007/s10701-011-9557-0

[12] : An Astrophysical Peek into Einstein’s Static Universe: No Dark Energy. *International Journal of Astronomy and Astrophysics*.2011;01(04) : 10.4236/ijaa.2011.14024183-19910.4236/ijaa.2011.14024

[13] : Deriving Friedmann Robertson Walker metric and Hubble’s law from gravitational collapse formalism. *Results in Physics*.2012;2: 10.1016/j.rinp.2012.04.00245-4910.1016/j.rinp.2012.04.002

[14] : Interpretational conflicts between the static and non-static forms of the de Sitter metric.*Sci Rep*.2012;2: 10.1038/srep0092392310.1038/srep0092323213359PMC3513772

[15] : ΛCDM COSMOLOGY THROUGH THE LENS OF EINSTEIN'S STATIC UNIVERSE, THE MOTHER OF Λ. *International Journal of Modern Physics D*.2013;22(03) : 10.1142/S021827181350012010.1142/S0218271813500120

[16] : Endoparasites in cattle nearby tribal areas of free-ranging protected areas of Tamil Nadu state.*J Parasit Dis*.2014;38(4) : 10.1007/s12639-013-0266-9429-3110.1007/s12639-013-0266-925320498PMC4185031

[17] : Energy of Einstein's Static Universe And Its Implications For The Lambda CDM Cosmology. *J.Cosmol.Astropart.Phys*.2013;

[18] Einsteinian Revolution's Wrong Turn: Lumpy Interacting Cosmos Assumed As Smooth Perfect Fluid, No Dark Energy, Eternal Universe?,Plenary Talk in New Results and Actual Problems in Particle.

[19] : Why the Big Bang Model does not allow inflationary and cyclic cosmologies though mathematically one can obtain any model with favourable assumptions. *New Astronomy*.2014;30: 10.1016/j.newast.2013.12.00246-5010.1016/j.newast.2013.12.002

[20] : When can an "Expanding Universe" look "Static" and vice versa: A comprehensive study. *International Journal of Modern Physics D*.2015;24(05) : 10.1142/S021827181550032710.1142/S0218271815500327

[21] : A diatribe on expanding space. Reference source

[22] : Criticism to Universal Big Bang. *Journal of Astrophysics & Aerospace Technology*.2016;04(01) : 10.4172/2329-6542.100012910.4172/2329-6542.1000129

[23] : On the Use of the Energy-Momentum Principle in General Relativity. *Physical Review*.1930;35(8) : 10.1103/PhysRev.35.875875-89510.1103/PhysRev.35.875

[24] : Relativity, Thermodynamics and Cosmology. *Nature*.1935;135(3423) : 10.1038/135935a0935-93610.1038/135935a0

[25] : The Energy-Momentum Problem in General Relativity.2003;

# Response

**Published:** 2020-09-21

**Authors:** Hartmut Traunmüller

**Affiliations:** Stockholm University, Sweden

Reviewer’s comment: 1. First, visibility of LSS has got nothing to do with the issue: “ light escapes faster than matter can move, (and we) ….are made of matter from this very source”.  When we observe any object say a table in the room, our eyes or our telescopes need to receive the light emanating from the surface of the table, and we do not require the matter constituting the table to strike our eyes or our telescopes. Thus it is immaterial whether matter of the table moves towards us with the speed of light or subluminal speed or with zero speed.

 Author’s response: The motion of the observed object is largely irrelevant, but I talk nowhere about it. Instead, it is crucial that, in order to see an event, the observer needs to be in a place where the light from the event has not yet passed. (You may think of a supernova explosion.) In the revised text, I have now made this explicit before telling that “we cannot reasonably be ahead of the light.” The latter becomes impossible soon after last scattering if the observer consists of matter that had its origin behind the LSS. Light escapes from there at c, while matter moves at v << c. The light has, thus, moved a longer distance than the matter of which we consist. The implication that the light must be reflected back to us or take a curved return path in order to be seen by us now is easy to understand.

 Reviewer’s comment: 2. Both the Model 1 & 2 mentioned by the author are flat models (k=0). However he mentions of “reflective boundary” in one case. There is no such different versions of the flat model, and the idea of a “reflective boundary” is incorrect and I am afraid shows basic misconceptions. In fact, much of the prolonged discussions are hardly comprehensible and I am afraid, results from confused thinking and misunderstanding. There just cannot be any boundary of the universe.

 Author’s response: I do not suggest *any* model to be correct. I discuss the Models 2 and 3 since either a reflective boundary or a curved return path could make the alleged last scattering visible to us in a Big Bang model. I hope this to have become clear by my response to the Reviewer’s first comment. Although Model 2, with its reflective boundary, is not a standard Big Bang model, it deserves to be said that it is hardly workable either and that it  shares some aspects with the inconsistent present standard model. (I would now rather say a little more about Model 3.)

 Reviewer’s comment: 2. If there is a homogeneous plasma, under ideal conditions, it will remain so during expansion or contraction irrespective of the strength of coupling between ionized matter and photons. However, the photons will get constantly scattered and remain tightly tied up with the plasma as long as the Thompson scattering cross section will be sufficiently larger than unity. During such a stage, a section of the plasma will not be able to see distant sections because photons from distant sections are not able to free stream.

 Author’s response: This is the situation *before* last scattering, which I do not comment. My paper is about what happens thereafter.

 Reviewer’s comment: 3. During expansion, at some stage, the density of the plasma will decrease so much that the plasma – photon scattering cross-section will drop below unity, and photons will be able to free stream. Suppose this happens when the radius of the plasma is R(t _0_). Then for t> t _0_, any section of the plasma, which may have condensed to neutral matter, lying beyond R(t) > R(t _0_), will be able to see the region beyond R(t _0_). And it would appear that all the light his receiving is emanating from the LSS: R(t _0_).

 Author’s response: At the end of the caption of Figure 1, I tell that the LSS remains visible until, very roughly, *η = *2 Gyr conformal time, *t = *2 Myr after the Big Bang. Thereafter, a reflection or a curved return path would be necessary.

 Reviewer’s comment: 4. Thus the LSS is sometimes called the cosmic photosphere, by analogy with the visible `surface' of the Sun where radiation produced by nuclear reactions is last scattered by the solar material. And contrary to what the author insists, visibility of the LSS has nothing to with whether universe is closed, where there could be return path of photons, or open/flat, where there cannot be any return path of photons.

 Author’s response: Visibility and invisibility of the LSS and its dependence on imaginable factors such as curvature and a reflective boundary is what I discuss in its essentials, and since the reviewer went off track already at the beginning of my description of the problem, it is not surprising that his conclusions here are sharply at variance with mine. I hope that this reviewer and readers in general will remain on track after the mentioned amendment.   

 Reviewer’s comment: 5. Having mentioned my above critique, let me tell that, yet there are reasons for introspection of some of the points raised by the author.

 Author’s response: The abundant remarks that follow here may be relevant in a wider frame but do not directly concern the simple reasoning in my paper.

# Peer review: Reviewer response for version 3

**DOI:** 10.5256/f1000research.27713.r67082

**Published:** 2020-08-06

**Authors:** Marcos C.D. Neves

**Affiliations:** 1 Physics Department, State University of Maringá, Maringá, Brazil

The paper proposes to study cosmology from a critical reading, presenting and discussing four models that are didactically presented throughout the text. The author makes an insufficient historical review, especially for the works that demonstrated that the temperature of radiation (or space) before the consolidation of the BB model occurred many years before 1960s until the "discovery" of Arno Penzias and Robert Wilson. A more precise historical path would be necessary utterly to consolidate the author's criticism of the current BB paradigm. 

 Although the author cites Assis & Neves (1995), it is necessary to point out that cosmologies not only of steady state as they were known, but cosmologies considering an universe infinite in space and in time that predicted values o temperatures of interstellar or intergalactic space with temperatures closer to that discovered by Penzias & Wilson that Gamow (greater than 50 K) or Dicke et al (greater than 30 K). 

 The names of the scientists linked to best previsions of the CMB or the temperature of space with no BB framework are Guillaume (Nobel Prize) at the end of 19th century, Eddington (the man responsible to Einstein win the Nobel Prize), Regener, Nernst (nobel Prize), Finlay-Freundlich, De Broglie (Nobel Prize), Max Born (Nobel Prize). In the documentary UNIVERSE, THE COSMOLOGY QUEST (directed by Randall Meyers, 2004), we can see these previsions based upon the Works of Assis & Neves (1993, 1999) and Peratt (1995). 

 This documentary is very important because give the opportunity by discordant voices of great scientists like: Geoffrey and Margaret Burbidge, Fred Hoyle, Halton Arp, Andre Assis, Jayant Narlikar, Jean-Claude Pecker and others.

 In this year we lost Margaret Burbidge, and seven years ago, Halton Arp. The dissappearances of these great scientists implies an impoverishment of the great debates about cosmology. Unfortunately, nowadays we are in an historical well-known sequence of the old greek astronomy: reinventing new epicycles by the same deferent circle (“ad hoc science”):  

  

- Invention of dark matter and dark energy to “explain” the acceleration of inflation; 
- The re-reading of the results gave by COBE’s observation; 
- The interpretation of the famous Hubble’s photo of the ultra deep field in Fornax constelation: all amateur astronomer knows that a photo of the night sky it is necessary a long exposure technique. It is the same by the Hubble. Old galaxies are less energetic (faint bright); contrary, Young galaxies are highly energetic. The interpretation of the "fact" given by the ultra deep field could represent the limitations of our telescopic technology. If we had a double or a triple time of the exposure, certainly, the photography will reveal old galaxies between young galaxies. But this assumption never is discussed in the scientific society because the BB paradigm is strongly consolidated.

We can quote here Imre Lakatos:  

 “The story is about an imaginary case of planetary misbehaviour. A physicist of the pre-Einsteinian era takes Newton's mechanics and his law of gravitation, (N), the accepted initial conditions, I, and calculates, with their help, the path of a newly discovered small planet, p. But the planet deviates from the calculated path. Does our Newtonian physicist consider that the deviation was forbidden by Newton's theory and therefore that, once established, it refutes the theory N? No. He suggests that there must be a hitherto unknown planet p' which perturbs the path of p. He calculates the mass orbit, etc., of this hypothetical planet and then asks an experimental astronomer to test his hypothesis. The planet p' is so small that even the biggest available telescopes cannot possibly observe it: the experimental astronomer applies for a research grant to build yet a bigger one.' In three years' time the new telescope is ready. Were the unknown planet p' to be discovered, it would be hailed as a new victory of Newtonian science. But it is not. Does our scientist abandon Newton's theory and his idea of the perturbing planet? No. He suggests that a cloud of cosmic dust hides the planet from us. He calculates the location and properties of this cloud and asks for a research grant to send up a satellite to test his calculations. Were the satellite's instruments (possibly new ones, based on a little-tested theory) to record the existence of the conjectural cloud, the result would be hailed as an outstanding victory for Newtonian science. But the cloud is not found. Does our scientist abandon Newton's theory, together with the idea of the perturbing planet and the idea of the cloud which hides it? No. He suggests that there is some magnetic field in that region of the universe which disturbed the instruments of the satellite. A new satellite is sent up. Were the magnetic field to be found, Newtonians would celebrate a sensational victory. But it is not. Is this regarded as a refutation of Newtonian science? No. Either yet another ingenious auxiliary hypothesis is proposed or. . .the whole story is buried in the dusty volumes of periodicals and the story never mentioned again.” (LAKATOS, I. The methodology of scientific research Programmes Philosophical Papers. NY: Cambridge University Press, 1989; p.16-17 ^1^) 

 Lakatos follows: 

 "This story strongly suggests that even a most respected scientific theory, like Newton's dynamics and theory of gravitation, may fail to forbid any observable state of affair. Indeed, some scientific theories forbid an event occurring in some specified finite spatio-temporal region (or briefly, a 'singular event ') only on the condition that no other factor (possibly hidden in some distant and unspecified spatio-temporal corner of the universe) has any influence on it. But then such theories never alone contradict a 'basic' statement: they contradict at most a conjunction of a basic statement describing a spatio-temporally singular event and of a universal non-existence statement saying that no other relevant cause is at work anywhere in the universe. And the dogmatic falsificationist cannot possibly claim that such universal non-existence statements belong to the empirical basis: that they can be observed and proved by experience. Another way of putting this is to say that some scientific theories are normally interpreted as containing a ceteris paribus clause:' in such cases it is always a specific theory together with this clause which may be refuted. But such a refutation is inconsequential for the specific theory under test because by replacing the ceteris paribus clause by a different one the specific theory can always be retained whatever the tests say. If so, the 'inexorable' disproof procedure of dogmatic falsificationism breaks down in these cases even if there were a firmly established empirical basis to serve as a launching pad for the arrow of the modus tollens: the prime target remains hopelessly elusive. And as it happens, it is exactly the most important, 'mature' theories in the history of science which are prima facie undisprovable in this way. Moreover, by the standards of dogmatic falsificationism all probabilistic theories also come under this head: for no finite sample can ever disprove a universal probabilistic theory; probabilistic theories, like theories with a ceteris paribus clause, have no empirical basis. But then the dogmatic falsificationist relegates the most important scientific theories on his own admission to metaphysics where rational discussion - consisting, by his standards, of proofs and disproofs - has no place, since a metaphysical theory is neither provable nor disprovable. The demarcation criterion of dogmatic falsificationism is thus still strongly antitheoretical."

  

- all the “re-writing” of the the theories of conservation (matter, energy, mainly); 
- all the predictions of the temperature of the space could be inherent to inflationary or not inflationary conception of the universe 
- all the predictions of the temperature of the space could be inherent to inflationary or not inflationary conception of the universe 

The author of the paper under analysis: “Does standard cosmology really predict the cosmic microwave background?”, as I pointed previously, presented 4 models of the “comprehension” of the Universe based upon the BB’s paradigm. I understood the aim of the author confrontates four scenarios to arrive a not accuracy of the results to validade the BB’s paradigm. Remembering Feyerabend: 

 "Einstein's first cosmological paper is a purely theoretical exercise containing not a single astronomical constant. The subject of cosmology itself for a long time found few supporters among physicists. Hubble the observer was respected, the rest had a hard time: Journals accepted papers from observers, giving them only the most cursory refereeing whereas our own papers always had a stiff passage, to a point where one became quite worn out with explaining points of mathematics, physics, fact and logic to the obtuse minds who constitute the mysterious anonymous class of referees, doing their work, like owls, in the darkness of the night. Is it not really strange', asks Einstein, 'that human beings are normally deaf to the strongest argument while they are always inclined to overestimate measuring accuracies?' - but just such an 'overestimating of measuring accuracies' is the rule in epidemiology, demography, genetics, spectroscopy and in other subjects." (FEYERABEND, P.K. Against Method. NY: Verso, 1993, p.239 ^2^)

 and, 

 Finally, the manner in which we accept or reject scientific ideas is radically different from democratic decision procedures. We accept scientific laws and scientific facts, we teach them in our schools, we make them the basis of important political decisions, but without ever having subjected them to a vote. Scientists do not subject them to a vote - or at least this is what they say - and laymen certainly do not subject them to a vote. Concrete proposals are occasionally discussed, and a vote is suggested. But the procedure is not extended to general theories and scientific facts. Modern society is 'Copernican' not because Copernicanism has been put on a ballot, subjected to a democratic debate and then voted in with a simple majority; it is 'Copernican' because the scientists are Copernicans and because one accepts their cosmology as uncritically as one once accepted the cosmology of bishops and cardinals. (FEYERABEND, P.K. Against Method. NY: Verso, 1993 ^2^) 

 To illustrate this report to support the criticism on the BB paradigm it is necessary to present the predictions of the temperature of space since Guillaume(table 1 - page 17, figure 2 - page 8 ^3^.

 It was published in 2004 a kind of “open letter”, by a team of dissents scientists, entitled “Bucking the Big Bang” in NEW SCIENTIST, presenting the great troubles present in the BB paradigm in the sense to support researches in concurrent theories of non-inflationary concept of Universe: 

 "The big bang today relies on a growing number of hypothetical entities, things that we have never observed-- inflation, dark matter and dark energy are the most prominent examples. Without them, there would be a fatal contradiction between the observations made by astronomers and the predictions of the big bang theory. In no other field of physics would this continual recourse to new hypothetical objects be accepted as a way of bridging the gap between theory and observation. It would, at the least, raise serious questions about the validity of the underlying theory. But the big bang theory can't survive without these fudge factors. Without the hypothetical inflation field, the big bang does not predict the smooth, isotropic cosmic background radiation that is observed, because there would be no way for parts of the universe that are now more than a few degrees away in the sky to come to the same temperature and thus emit the same amount of microwave radiation. Without some kind of dark matter, unlike any that we have observed on Earth despite 20 years of experiments, big-bang theory makes contradictory predictions for the density of matter in the universe. Inflation requires a density 20 times larger than that implied by big bang nucleosynthesis, the theory's explanation of the origin of the light elements. And without dark energy, the theory predicts that the universe is only about 8 billion years old, which is billions of years younger than the age of many stars in our galaxy. What is more, the big bang theory can boast of no quantitative predictions that have subsequently been validated by observation. The successes claimed by the theory's supporters consist of its ability to retrospectively fit observations with a steadily increasing array of adjustable parameters, just as the old Earth-centred cosmology of Ptolemy needed layer upon layer of epicycles."

 (Eric Lerner, Bucking the Big Bang. New Scientist, 22 May, 2004, in: https://www.newscientist.com/article/mg18224482-900-bucking-the-big-bang/) ^4^

 The epicycle is a metaphor regards to the ancient greek practical in astronomic model to “save the phenomenon”, what means that where the prediction of the planet fail, another epicycle is necessary upon to be placed on the first epicycle to adjust the measurement (see image). 

 Jayant Narlikar, the great indian astrophysicist, says in the documentary UNIVERSE, THE COSMOLOGY QUEST says: “however what it happens along the years is that always when the observations are not agree of the BB previsions, the theory creates a new assumption that is not all of tested or based in a conventional physics and simply assumes that must be true”. This is the same that was expressed by Imre Lakatos in his story about an hypothetical perturbing planetIn nowadays, the enigmatic nature of CMB or the ad hoc assumption of dark matter or dark energy can explained the nature of Cosmology as a big speculative science and, by principle, open to several hypothesis, theories and models, but …, unfortunately this is not the case. 

 Remembering an important theme in a science history investigation: 

 "The earliest estimation of a temperature of “space” known to us is that of Guillaume (1896). It was published in 1896, prior to Gamow’s birth (1904). Here we quote from this paper (English translation by C. Roy Keys): “Captain Abney has recently determined the ratio of the light from the starry sky to that of the full Moon. It turns out to be 1/44, after reductions for the obliqueness of the rays relative to the surface, and for atmospheric absorption. Doubling this for both hemispheres, and adopting 1/600000 as the ratio of the light intensity of the Moon to that of the Sun (a rough average of the measurements by Wollaston, Douguer and Zöllner), we find that the Sun showers us with 15,200,000 time more vibratory energy than all the stars combined. The increase in temperature of an isolated body in space subject only to the action of the stars Page 80 APEIRON Vol. 2 Nr. 3 July 1995 will be equal to the quotient of the increase of temperature due to the Sun on the Earth’s orbit divided by the fourth root of 15,200,000, or about 60. Moreover, this number should be regarded as a minimum, as the measurements of Captain Abney taken in South Kensington may have been distorted by some foreign source of light. We conclude that the radiation of the stars alone would maintain the test particle we suppose might have been placed at different points in the sky at a temperature of 338/60 = 5.6 abs. = 207º.4 centigrade. We must not conclude that the radiation of the stars raises the temperature of the celestial bodies to 5 or 6 degrees. If the star in question already has a temperature that is very different from absolute zero, its loss of heat is much greater. We will find the increase of temperature due to the radiation of the stars by calculating the loss using Stefan’s law. In this way we find that for the Earth, the temperature increase due to the radiation of the stars is less than one hundred thousandth of a degree. Furthermore, this figure should be regarded as an upper limit on the effect we seek to evaluate.” Of course, Guillaume’s estimation of a 5-6 K blackbody temperature may not have been the earliest one, as Stefan’s law had been known since 1879. Moreover, it is restricted to the effect due to the stars belonging to our own galaxy"

 in: History of the 2.7 K Temperature Prior to Penzias and Wilson by Assis & Neves, Apeiron Vol. 2 Nr. 3 July 1995 Page 79-84] ^5^. 

 Geoffrey Burbidge, wrote also: 

 "We had a good discussion of various issues relating to cosmology and there has been a clear division of perceptions of what is considered important evidence. On the one side, the conventional one, we have heard the very detailed evidence of CMBR and high redshift supernovae, evidence that is popularized in the phrase “concordance cosmology.” The Universe according to this view went through an inﬂationary phase, had an era of nucleosynthesis and then had the surface of last scattering when the radiation background became decoupled from matter. The package comes with a large part of the matter energy (around 75%) being dark and hitherto unknown, a substantial part of strange kind of matter (21%) and only around 4% of ordinary matter that we are familiar with. Once you believe all of these ideas, you feel convinced that the cosmological problem is all but solved. On the other side, some of us have been increasingly worried at what appears to be anomalous evidence, evidence that does not ﬁt into the standard picture just mentioned. Even the very basic Hubble law applied to QSO redshifts seems to be threatened if one takes the evidence on anomalous redshifts seriously. In the 1970s when Chip Arp ﬁrst started ﬁnding such examples, he was told that these were exceptions and that he should ﬁnd more. He has been doing just that and his cases now include not just optical sources but also radio and X-ray sources. Then there is the evidence of periodicities of redshifts that has not gone away with larger samples. As I discussed, even the gamma ray burst sources appear to show the effect. While there are many things that we do not understand we believe that this cosmogonical evidence ﬁts well into the cyclic universe scheme. The contrast between the two perceptions gets further highlighted when one notices the large number of speculative concepts that have gone into the standard paradigm: The nonbaryonic dark matter, dark energy, phase transitions at energy well beyond the range tested in the laboratory, etc. These relate to parts of the Universe that will remain forever unobservable and whose physics will remain forever untested in the laboratory. However, without making these assumptions the theory fails. The fact is that we do not know how galaxies form, and for them to form in a big-bang Universe it is necessary to invoke initial density ﬂuctuations and a large amount of nonbaryonic matter to make them condense. On the other hand, the anomalous evidence ignored by the conventional cosmologists is real, right on our doorstep, and well observable. Surely we need to probe it further and in a way that will enable us to understand if any new physics is needed here. It is unfortunate that the majority of the cosmology community chooses to ignore all of this evidence in the hope that it will go away."[Burbidge, G. Panel Discussion. In: Pecker, J-C; Narlikar, J. CURRENT ISSUES IN COSMOLOGY. Cambridge University Press, 2006, p. 237-238] ^6^

 The conclusion of the paper under analysis could be enriched by the Edwin Hubble’s arguments when he detected galaxies in recessive motion at a “incredible” speed at 0,14 c . If Hubble had observed quasars in the 1920s he would never have come to his law:  v = H . d(Graph in DVD 2 ^7^. 

 Hubble (apud Assis & Neves, 1995 ^5^) wrote at the end years of his life: 

 "Light may lose energy during its journey through space, but if so, we do not yet know how the energy loss can be explained. The disturbing features are all introduced by the recession factor, by the assumption that red-shifts are velocity-shifts. The departure from linear law of redshifts, the departure from uniform distribution, the curvature necessary to restore homogeneity, the excess material demanded by the curvature; each of these is merely the recession factor in another form. These elements identify a unique model among the array of possible expanding worlds, and, in this model, the restriction in time-scale, the limitation of spatial dimensions, the amount of unobserved material, is each equivalent to the recession factor. On the other hand, if the recession factor is dropped, if redshifts are not primarily velocity-shifts, the picture is simple and plausible. There is no evidence of expansion and no restriction of the time-scale, no trace of spatial curvature and no limitations of spatial dimensions. We seem to face, as once before in the days of Copernicus, a choice between a small, finite universe, and a universe indefinitely large plus a new principle of nature."

 To conclude, the author of the present paper used correctly Lakatos to emphasize the serious limitation of the BB’s paradigm. Jean-Claude Pecker, the famous astrophysicist at the Collège de France, in a speech in the documentary UNIVERSE, THE COSMOLOGY QUEST afirmed:  

 "In August 1952 we have a Meeting of the International Astronomic Union in Rome and we were received the Pope Pio XII. And Pio XII made an address to the astronomers and this address was very clear, and he said: “oh, the BB is the Fiat lux! This is beautiful that Astronomy proves this and this , etc, etc…” I always an herectic of this all things. I didn’t believe in any God and when I see “Fiat lux” and the BB associated each other I was suspicious since the beginning and I forgot the BB. Those distant things about which physics was very vague were difficult to observe … but let’s face: in all the history of Astronomy from years and years, centuries and centuries, the progress came from observations and confrontations of these new observations and in the past theories sometimes contradictions, sometimes confirmations. Frequently these contradictions lead us to a progress and change in the theories. But this is not occurring [today]. So, actually, the 3K radiation for me don’t have any cosmological value. It is observed and occur in any cosmology we can predict the radiation of 3K. So, it is not the prove of any specific cosmology. We have to match what is observed. We have an observable universe that is made of stars and galaxies that are very far away and that is all. Radiation of 3K can be thought as of local origin ... beyond that, I think, it is a wild extrapolation and whatever it is, and the physics that we could imagine as being there is based on nothing because we have no tests available to verify over there."

 The author of the paper *Does standard cosmology really predict the cosmic microwave background?* wrote a difficult text by its intrinsic epistemological nature. All the quotations and discussion that I made above were to clarify the needs to put contemporary cosmology in a great and crucial debate between scientists with different worldviews,  theories and models as well as to an inflationary or to a non-inflationary universe (infinite in space and in the time). 

 Last, but not least, and rewriting Feyerabend, in Against the Method: our science is ‘BigBangnian’ because the scientists are ‘BigBagnian’ and because one accepts their cosmology as uncritically as one once accepted the cosmology of bishops and cardinals.

Is the topic of the opinion article discussed accurately in the context of the current literature?

Yes

Are arguments sufficiently supported by evidence from the published literature?

Partly

Are all factual statements correct and adequately supported by citations?

Yes

Are the conclusions drawn balanced and justified on the basis of the presented arguments?

Yes

Reviewer Expertise:

NA

I confirm that I have read this submission and believe that I have an appropriate level of expertise to confirm that it is of an acceptable scientific standard.

## References

[1] : The Methodology of Scientific Research Programmes. *Cambridge University Press*.1989;16-17Reference source

[2] : Against the Method. *Verso Books*.1993;

[3] : Plasma Astrophysics and Cosmology. *Springer Netherlands*.1995; Reference source

[4] : Bucking the Big Bang. *New Scientist*.2004; Reference source

[5] : History of the 2.7 K Temperature Prior to Penzias and Wilson. *Apeiron*.1995;2:79-84Reference source

[6] Current Issues in Cosmology.2006; 10.1017/CBO978051160702810.1017/CBO9780511607028

[7] : Universe, The Cosmology Quest. *Floating World Films*.2004; Reference source

# Response

**Published:** 2020-08-12

**Authors:** Hartmut Traunmüller

**Affiliations:** Stockholm University, Sweden

Author: This review does not really comment my line of reasoning. My basic criticism is this: In a flat, reflection-free Big Bang universe, the radiation from an assumed last scattering surface will soon after its emission have escaped altogether from the expanding matter-filled region and so become invisible. This happens when the time it takes light to traverse the last scattering surface has passed (in conformal/comoving terms, see Figure 1). Such a model fails entirely to explain the observed 2.7 K blackbody radiation and its apparent homogeneity. It predicts 0 K here and now.

 Reviewer 2: A more precise historical path would be necessary utterly to consolidate the author's criticism of the current BB paradigm. 

 Author: An improved account for the history of the disclosed error would indeed be desirable. It began with Tolman who (in 1931) considered the entropy of the universe as a whole and (in 1934) over-generalized the validity of his initial homogeneity assumption to expanding universes such as described by Friedman, Lemaître, and Robertson, and to oscillating ones. Gamow actually escapes my criticism because his universe was dense and positively curved (my model 3). Dicke, Peebles, Roll et al. (1965) followed Tolman in his overgeneralization and this error became an established tradition. I intend to complete the information in the next revision of my text in this sense, but the problem that errors like this one can pass unnoticed and become conventionalized among scientists deserves separate and deeper studies.

 Author: The reviewer adds many words about various further aspects of the BB paradigm that have been criticized by himself and by others, but which are marginal to my line of reasoning. I will stick to this line, but I can go one step in the reviewer’s direction and mention a further deficiency that makes Big Bang cosmology not just philosophically questionable, but outright untenable, viz. irrational if stuck to: In pondering what it is that participates in the expansion of the universe, astronomers have come to the conclusion that everything up to the size of galaxy clusters must be exempted. Only the voids between these clusters are free to expand. Under this premise, the matter density within the universe could never have been higher than it uses to be within galaxy clusters – never as high as assumed at the alleged event of last scattering.

# Peer review: Reviewer response for version 2

**DOI:** 10.5256/f1000research.26661.r64257

**Published:** 2020-06-08

**Authors:** Indranil Banik

**Affiliations:** 1 Helmholtz Institute for Radiation and Nuclear Physics (HISKP), University of Bonn, Bonn, Germany

The author's model 4 (supposedly representing the standard model) does not adequately represent the standard picture. In particular, the author has made the so-called chronogonic assumption that the cosmic scale factor a(t) was never smaller than now. But in the standard model, it has increased by a factor of 1090 since the time of last scattering.

 Regarding the photons having a constant co-moving number density, this is correct - but the author still says it is wrong. This is because the author assumes some sort of finite Universe, with a single flash occurring 13.8 Gyr ago and illuminating some region outside which there is nothing. This is evident from Figure 1 and the authors statement "there is nothing to be gained from its non-existent (or at least empty) outside" - in the standard picture, there is no outside. Rather, the standard picture is that an infinitely large Universe appeared instantaneously 13.8 Gyr ago, and then a(t) started rising rapidly. **All of space was brought into existence by the Big Bang.** This is why in co-moving terms photon losses from some region are always (on average) balanced by photons gained from elsewhere. There is also the additional redshifting of photons by which they lose energy individually, but certainly the co-moving number density of photons should remain the same.

 In Figure 1, this leads to the author only considering photons emitted from one point on the last scattering surface. In reality, what would be more useful is to draw two more multiple orange last scattering surface (LSS) cones translated along x, such that they pass through our spacetime location (at the top). The apexes would be where the dotted blue lines intersect with (almost) zero conformal time. This would more accurately represent what happened in the Big Bang according to standard cosmology, and also address the issue of why GN-z11 is currently visible. It is well known that this does lead to a causality problem in that all these LSS cones have an apex at widely separated points, so shouldn't have been in causal contact even earlier - and thus should have a different temperature. This problem is addressed by the inflationary hypothesis.

 Another issue is that some sort of confinement is needed to get blackbody radiation, but the author mistakenly assumes that the whole Universe must have some sort of reflective boundary. In reality, the confinement arises because the early Universe was opaque because neutral hydrogen atoms did not exist yet, and electrons have a large Thomson scattering cross section. So photons had only a short mean free path/travel time between absorptions. This is really the origin of the blackbody spectrum - not the walls of the Universe, which would be more analogous to terrestrial experiments involving a sealed-off room.

 At the end of page 7, the author states without justification that the visibility of high-z galaxies has not been reconciled with a hot Big Bang. There is no problem with this in the standard context - the galaxy would have been relatively close to us, but in an expanding universe, light from it would only just have been able to keep pace with the ever increasing distance remaining to the Earth. Most of the progress would be achieved at late times when the Hubble parameter was lower. The total travel time would be almost 13.8 Gyr. There is nothing unusual about this, in an infinite Universe. The problem with the authors's logic is that a finite Universe is assumed, which would indeed cause very serious problems.

 Although the last part on the sociological aspects is reasonably well done, I strongly recommend the author delete this last bit: but it is, of course, even more fundamental to respect reason at all.

 In conclusion, the fundamental assumptions underpinning the article are completely incorrect for the reasons I have explained. Therefore, I still have to recommend rejection of the revised article, and also recommend that in future other reviewers be used in order to get a second opinion. My opinion is that it is not possible to edit the article in such a way that it can ever be approved, since the only novel claim in the article is that all standard cosmologists over the past 80 or so years have made a basic blunder - but it is completely clear to me that the blunder is on the part of the author, who has not understood the most basic aspects of the standard hot Big Bang cosmological model.

Is the topic of the opinion article discussed accurately in the context of the current literature?

Yes

Are arguments sufficiently supported by evidence from the published literature?

No

Are all factual statements correct and adequately supported by citations?

No

Are the conclusions drawn balanced and justified on the basis of the presented arguments?

No

Reviewer Expertise:

Modified Newtonian Dynamics (MOND). I felt I was able to assess all aspects of the article, as I work on a non-mainstream cosmological model and need to pay close attention to issues like the cosmic microwave background radiation and cosmology, which remains a little uncertain in MOND

I confirm that I have read this submission and believe that I have an appropriate level of expertise to state that I do not consider it to be of an acceptable scientific standard, for reasons outlined above.

# Response

**Published:** 2020-06-27

**Authors:** Hartmut Traunmüller

**Affiliations:** Stockholm University, Sweden

Reviewer’s comment: The author's model 4 (supposedly representing the standard model) does not adequately represent the standard picture. In particular, the author has made the so-called chronogonic assumption that the cosmic scale factor a(t) was never smaller than now. But in the standard model, it has increased by a factor of 1090 since the time of last scattering.

 Author’s response: I think I made it clear enough under *Model 4* that two incommensurable models are confounded with each other in the present standard model, but I was not explicit enough about their incompatible characteristics. The basic model is the traditional, ‘cosmogonic’ FLRW Big Bang model, in which a(t) has increased as the reviewer says. The ‘chronogonic’ supplementary one has its origin in the assumption of a homogeneous universe. This and the characteristics of both models are now made explicit in the first passage under *Model 4*. Under *Model 1*, I have inserted a new passage that prepares the reader for this.

 Reviewer’s comment: Regarding the photons having a constant co-moving number density, this is correct - but the author still says it is wrong. This is because the author assumes some sort of finite Universe, with a single flash occurring 13. 8 Gyr ago and illuminating some region outside which there is nothing. This is evident from Figure 1 and the authors statement “there is nothing to be gained from its non-existent (or at least empty) outside” - in the standard picture, there is no outside. Rather, the standard picture is that an infinitely large Universe appeared instantaneously 13. 8 Gyr ago, and then a(t) started rising rapidly.

 Author’s response: This is not the standard picture. In the literature I refer to, the Big Bang universe is **finite** and expanding, and this is still the basic model. A universe that was formerly large enough for us to see light that was emitted almost 13.8 Gyr ago exists ***only*** in the supplementary chronogonic model, while the rising a(t) exists ***only*** in the cosmogonic model. For a rational analysis, these models need to be considered each on its own terms – also for knowing what is meant by “outside” and by

 Reviewer’s comment: **All of space was brought into existence by the Big Bang.**

 Author’s continued response: In the cosmogonic model, this is the space above the V-shaped future light cone of the Big Bang in Figure 1. In the chronogonic model, the existing space is that above the abscissa. The reception of radiation from any sources below the golden band in Figure 1 is only compatible with the latter model. If reason is to rule and one accepts this model, one has to reject the Big Bang model (also vice versa).

 Reviewer’s comment: This is why in co-moving terms photon losses from some region are always (on average) balanced by photons gained from elsewhere. There is also the additional redshifting of photons by which they lose energy individually, but certainly the co-moving number density of photons should remain the same.

 Author’s response: If it could occur at all, such balancing would *only* be possible in the chronogonic model, while co-moving terms apply *only* in the cosmogonic one, in which this “elsewhere” does not exist. However, the chronogonic model does not give rise to a homogeneous blackbody radiation at all. I think this should be clear from my text, and also that **we** are not in a position within the golden band. To make this clearer, the caption of Figure 1 is now converted from telegraph style into plain English.

 Reviewer’s comment: In Figure 1, this leads to the author only considering photons emitted from one point on the last scattering surface. In reality, what would be more useful is to draw two more multiple orange last scattering surface (LSS) cones translated along x, such that they pass through our spacetime location (at the top). The apexes would be where the dotted blue lines intersect with (almost) zero conformal time. This would more accurately represent what happened in the Big Bang according to standard cosmology, and also address the issue of why GN-z11 is currently visible.

 Author’s response: The golden band in Figure 1 represents all radiation from the LSS in a flat Big Bang universe, i. e. , from **all** points on the LSS. It covers the whole region traversed by the radiation (an expanding shell with a thickness of slightly less than 2 Glyr comoving distance). It is my aim to disclose the deficiency – not to obfuscate it in any way.

 Reviewer’s comment: It is well known that this does lead to a causality problem in that all these LSS cones have an apex at widely separated points, so shouldn't have been in causal contact even earlier - and thus should have a different temperature. This problem is addressed by the inflationary hypothesis.

 Author’s response: I remain silent about this well-noted problem, which is caused by the homogeneity of the CMB, allegedly emitted from the LSS, because the models I analyze do not even offer a rational explanation for the very visibility of the LSS.

 Reviewer’s comment: Another issue is that some sort of confinement is needed to get blackbody radiation, but the author mistakenly assumes that the whole Universe must have some sort of reflective boundary.

 Author’s response: I consider variations of four models. A reflective boundary is only assumed in model 2, and I judged all four models a untenable. However, I **point out** that a non-reflective flat Big Bang universe offers no possibility for us to see the LSS. This follows logically from what we know about the propagation of light and the motion of matter. A chronogonic universe would allow us to see something at otherwise excessive distances, but I point out that it does not give rise to anything like the LSS.

 Reviewer’s comment: In reality the confinement arises because the early Universe was opaque because neutral hydrogen atoms did not exist yet, and electrons have a large Thomson scattering cross section. So photons had only a short mean free path/travel time between absorptions. This is really the origin of the blackbody spectrum - not the walls of the Universe, which would be more analogous to terrestrial experiments involving a sealed-off room.

 Author’s response: I did not subject this alleged reality to any criticism at all. My criticism concerns what the model predicts to happen **after **the alleged event of last scattering, which I took as given in my reasoning.

 Reviewer’s comment: At the end of page 7, the author states without justification that the visibility of high-z galaxies has not been reconciled with a hot Big Bang.

 Author’s response: It has not been reconciled with a Big Bang since these galaxies are located **outside the space a Big Bang model offers. **I still think I had made this clear enough on the preceding pages.

 Reviewer’s comment: There is no problem with this in the standard context - the galaxy would have been relatively close to us, but in an expanding universe, light from it would only just have been able to keep pace with the ever increasing distance remaining to the Earth. Most of the progress would be achieved at late times when the Hubble parameter was lower. The total travel time would be almost 13. 8 Gyr. There is nothing unusual about this, in an infinite Universe. The problem with the authors's logic is that a finite Universe is assumed, which would indeed cause very serious problems.

 Author’s response: It is not the *finiteness* but the former *smallness* of the Big Bang universe that causes the problem. When there is no other way out, the mainstream accepts that the universe was at least as large as it is now (not necessarily infinite) already 13. 8 Gyr ago. However, this is blatantly irrational if a Big Bang model, in which the universe was much smaller before, is used and crucial for explaining other aspects of the universe and the CMB.

 Reviewer’s comment: Although the last part on the sociological aspects is reasonably well done, I strongly recommend the author delete this last bit: but it is, of course, even more fundamental to respect reason at all.

 Author’s response: To me, this statement appears to the point. I see nothing controversial in it. It expresses the generalized lesson of this article – which admittedly can be hard for the wrongly indoctrinated.

 Reviewer’s comment: In conclusion, the fundamental assumptions underpinning the article are completely incorrect for the reasons I have explained. Therefore, I still have to recommend rejection of the revised article, and also recommend that in future other reviewers be used in order to get a second opinion. My opinion is that it is not possible to edit the article in such a way that it can ever be approved, since the only novel claim in the article is that all standard cosmologists over the past 80 or so years have made a basic blunder - but it is completely clear to me that the blunder is on the part of the author, who has not understood the most basic aspects of the standard hot Big Bang cosmological model.

 Author’s response: I am not aware of any accusation that I did not respond to adequately. It is, of course, more reasonable to believe that it is me, an outsider, who has made a blunder than that the whole community of professionals, which includes several Nobel laureates, all could have repeated the same blunder for many decades. However, this is not a physical argument. It is the natural prejudice that I hoped to bypass by submitting my article to F1000Research. I confirm that I have not understood the standard cosmological model, but I claim that nobody else can have understood it either, because it defies rationality.

 I would, anyway, like to express my gratitude to Dr Indranil Banik for having accepted the role of a public reviewer of my article at all. His comments have brought me to make several clarifying amendments.

# Peer review: Reviewer response for version 1

**DOI:** 10.5256/f1000research.24751.r62422

**Published:** 2020-04-29

**Authors:** Indranil Banik

**Affiliations:** 1 Helmholtz Institute for Radiation and Nuclear Physics (HISKP), University of Bonn, Bonn, Germany

The following article claims to raise serious conceptual problems with the standard cosmological model:

 I have to recommend that the article be rejected. First of all, the discussion section is offensive - as a researcher working on non-mainstream ideas, I can understand the sometimes difficult struggle when challenging the mainstream paradigm. But to suggest that I am recommending rejection in order to protect my career is extremely offensive, when my career in fact relies on challenging the mainstream view. The referee might like to know this before dismissing my rejection as a sign of anything other than scientific invalidity of his ideas. But I agree that it is occasionally possible for articles to be rejected which are actually correct, because the referee is protecting personal interests. This is certainly not as common as the author makes out, and indeed I have had generally respectful discussions with mainstream cosmologists despite viewing the Universe very differently. For such a (hopefully) polite discussion, the author may like to watch this debate:

https://www.eso.org/sci/meetings/2020/Cosmic-Duologues.html

 Regarding the article itself, the main problem is the author has not understood the basics of the Big Bang model in which there is not an explosion in space, but an expansion of space. In this model, the universe is infinite and almost homogeneous at early times. In co-moving co-ordinates, this expansion is cancelled out and you would just see a static Universe. Suppose a flash of light is emitted from every location at the same time. Photons in some region would of course be moving at c, thus leaving through the boundary - but other photons would enter. The number density of photons would thus remain the same in co-moving co-ordinates. There is no inconsistency here. It explains why we expect to measure the same co-moving number density of primordial photons today as there was at the time of last scattering.

 Regarding the issue of where the surface of last scattering is, the author should simply consider a photon that has been travelling at c for a Hubble time in a straight line. The result is at some distance, independent of direction - so the surface is a sphere. However, this is not a real surface - it is just the locus of points from which photons will later hit the Earth exactly 13.8 Gyr later. At the time of emission, nothing whatsoever is special about material in this surface. The whole sphere may well be much larger than 380 kly, which is somewhat non-intuitive since the age of the universe was (in this model) only 380 kyr then.

 The author raises an important point about how the Universe was in thermal equilibrium at early times. This is related to the horizon problem, which - as the author points out - is thought to be resolved by inflation. Briefly, the idea is that the Universe was small for an extended period of time, during which it was in causal contact and thus reached thermal equilibrium. The particle horizon then expanded faster than c due to a period of accelerated expansion similar to what we are experiencing now, causing that photons reaching us from different sides of the sky have the same temperature. The author then goes on to unscientifically attack the hypothesis of inflation - indeed, it is well known that this is not confirmed. However, the author does not raise any substantive arguments against the inflation model, and does not propose his own ideas.

 Of course, if the mainstream idea was that the Universe was 380 kly wide at some time 13.8 Gyr ago and that every location within it emitted photons at that moment (almost) equally in all directions, then it is clear that these photons would be unobservable now - depending on what happens at the edge. However, the Universe is not thought to have any such edge, and is considered to be infinite. It is certainly the case that light emitted from close to us would no longer be observable, so what we see as the cosmic microwave background (CMB) must have originated rather far away, on the surface of last scattering. The longer we wait, the further away this surface is - though it always corresponds to the same moment in the history of the universe. There will never be a moment when there is no CMB altogether (barring absorption of all its photons), because one can always imagine reversing the arrow of time and integrating a trajectory moving away from us at c in a contracting universe. The photon will have some well-defined location at the moment of last scattering, so this point will be part of the surface of last scattering to such an observer. No special reflective surface is required, and indeed no new assumptions are needed at all to explain the observability of the CMB. Of course, explaining its detailed properties remains a challenge given other constraints e.g. the Hubble tension. But the particular criticism of the standard cosmological model raised by the author is completely erroneous. Therefore, this manuscript must be rejected - even though I do consider that the same is true for the standard cosmological model as a whole, which does indeed contain many hypothetical ingredients that I am not at all a fan of & have publicly spoken against on several occasions e.g. here:

https://www.youtube.com/watch?v=PYVC0VtmpDg

Is the topic of the opinion article discussed accurately in the context of the current literature?

Yes

Are arguments sufficiently supported by evidence from the published literature?

No

Are all factual statements correct and adequately supported by citations?

No

Are the conclusions drawn balanced and justified on the basis of the presented arguments?

No

Reviewer Expertise:

Modified Newtonian Dynamics (MOND) I felt I was able to assess all aspects of the article, as I work on a non-mainstream cosmological model and need to pay close attention to issues like the cosmic microwave background radiation and cosmology, which remains a little uncertain in MOND

I confirm that I have read this submission and believe that I have an appropriate level of expertise to state that I do not consider it to be of an acceptable scientific standard, for reasons outlined above.

# Response

**Published:** 2020-05-14

**Authors:** Hartmut Traunmüller

**Affiliations:** Stockholm University, Sweden

Reviewer's comment: I have to recommend that the article be rejected. First of all, the discussion section is offensive - as a researcher working on non-mainstream ideas, I can understand the sometimes difficult struggle when challenging the mainstream paradigm. But to suggest that I am recommending rejection in order to protect my career is extremely offensive, when my career in fact relies on challenging the mainstream view. The referee might like to know this before dismissing my rejection as a sign of anything other than scientific invalidity of his ideas.

 Author's response: This review (there is no separate referee) emphasizes something other on the preceding lines. It has probably to do with my mentioning, in the next to last passage of the article, of a good  reason *editors* might have for rejecting certain manuscripts prior to review. In the same passage, I suggest a milder, equally rational explanation - one that applies beside editors also to *reviewers *and definitely also to myself in many analogous situations.

 Reviewer's comment: But I agree that it is occasionally possible for articles to be rejected which are actually correct, because the referee is protecting personal interests. This is certainly not as common as the author makes out,

 Author's response: This is common in certain cases, also self-censorship. To make it clear in which cases, I have inserted a reference to Lakatos, who had observed that the “hard core” of “research programmes” (such as mainstream cosmology) is beyond criticism.

 Reviewer's comment: the main problem is the author has not understood the basics of the Big Bang model in which there is not an explosion in space, but an expansion of space.

 Author's response: In the next to last passage of the *Introduction*, in which I summarize the characteristics of a Big Bang model that are essential here, I told that the expansion uses to be ascribed to “space”, i.e., to an expansion *of* space. An expansion *in* space was nowhere implied, but at the end of the second passage under *Model 4*, I have now inserted a parenthetic phrase “or at least empty” after “non-existent” only for telling that this related distinction makes no difference in this context.

 Author's response to the Reviewer's remaining comments: These have brought me to underline the fact that, in a Big Bang universe, it is not the case that blackbody radiation lost from a region is balanced by an equal amount gained from outside and, above all, to extend the description and analysis of the present standard model (new text under *Model 4*) as summarized in the "Update text". I kindly ask the reviewer for a new evaluation.
