# Supplementary material for: Does standard cosmology really predict the cosmic microwave background?
Source: F1000Res. 2021 Feb 19;9:261. Originally published 2020 Apr 16. [Version 5] doi: 10.12688/f1000research.22432.5 (PMC8127010; doi:10.12688/f1000research.22432.5)
Supplement: Peer review history

# Response

**Published:** 2020-12-18

**Authors:** Hartmut Traunmüller

**Affiliations:** Stockholm University, Sweden

Reviewer’s comment: The author assumes that at the time of recombination, there was only matter inside a volume with a 0.95 Gly comoving radius, so that light released after recombination is only emitted from within this volume. This is incorrect. 

 Author’s response: I assume this of a proper Big Bang universe, because ≈ 0.95 Gly is the comoving radius such a universe is calculated to have at the time of recombination, and there was *nothing* outside it at that time (not even a physical vacuum). In a new second passage under *Model 4*, it is now clarified how the standard model deviates from this.

 Reviewer’s comment: Despite having read Ryden's 'Introduction to Cosmology' the author ignores that matter is assumed to exist beyond the horizon: 'The stars *beyond some finite distance, called the horizon distance*, are invisible to us because their light hasn't had time to reach us yet' (Ryden 'Introduction to Cosmology' 2016, p. 10) ^1^.

 Author’s response: This quotation is taken from a passage (end of section 2.1) in which Olbers’ paradox is discussed. It refers to our observations in this context and not to what a Big Bang model predicts. The statement is, instead, in line with an Expanding View model.

 Reviewer’s comment: As a result of this misconception, the author incorrectly plots a short “red horizontal dash” in Fig. 1. But why would matter only be created within a given comoving radius from us? (Author: not only from our galaxy, but from any other galaxy as well!)

 Author’s response: The answer is obvious enough: because the Big Bang universe was no lager then – no wider than the short red horizontal dash.

 The reviewer consistently misinterprets what I say about a proper flat Big Bang model as incorrect statements about the standard model, in which such a model is supplemented with a *contradictory* model. I did point out the conflict between the constituent models in the first two passages under *Model 4*. The new second passage inserted there is intended to prevent the misinterpretation. It is only the contradictory supplement that allows anything to exist outside the proper Big Bang universe, i.e., below the golden V-shaped band in my Figure 1.

 As I explain in the first passages under *Model 4*, in standard cosmology, which is taught by Ryden, an “Expanding Universe” (Big Bang) model is supplemented with a contradictory “Expanding View” model.  I have now made it explicit that I had to coin the attributes “Expanding View” and “chronogonic” myself (inspired by correspondence with Barbara Ryden). In the literature, the supplementary model uses to be invoked informally, without being named. Together with the common but misleading practice of referring to the entire standard model as a Big Bang model, this dampens the awareness about the presence and contradictoriness of the supplement, as demonstrated by the comments from reviewers I. Banik and L. Marmet.

 The calculations of densities, redshifts, temperatures, etc. are still done in a Big Bang model whose comoving radius was ≈ 0.95 Gly when it became transparent. Consider what Ryden says in section 2.5 of her book: “The blackbody radiation that fills the universe today can be explained as a relic of the time when the universe was sufficiently hot and dense to be opaque. However, at the time the universe became transparent, its temperature was 2970 K. The temperature of the CMB today is 2.7255 K, a factor of 1090 lower. The drop in temperature of the blackbody radiation is a direct consequence of the expansion of the universe. Consider a region of volume *V *that expands at the same rate as the universe, so that *V *∝ a( *t*) ^3^. The blackbody radiation in the volume can be thought of as a photon gas with energy density *ε _γ_*= *αT* ^4^. ... The photon gas within our imaginary box follows the laws of thermodynamics; ...” Some of this and a reference to the corresponding section in the book is now inserted.

 Here, a Big Bang model is described, and the imaginary box does not exist in nature. Despite this, the calculations are done as if it was present. Ryden here merely follows a tradition, but this is the cardinal blunder I mention in the second passage under *Model 2*. Since there is actually no such box, there is no way for any relic radiation to remain within the volume *V *∝ a( *t*) ^3^ that is represented by the LSS-wide region between the two dashed vertical lines in my Figure 1. The  radiation cannot fail to escape from this region at *c* (within the golden V). Since this is missed in Ryden’s description, the model is flawed, but before being supplemented, it is still a Big Bang model in which nothing at all exists below the golden V. (Approximately this is now said under *Model 4* and contrasted with Tolmans approach that defies the Big Bang model already at this point.)

 Reviewer’s comment: Near the end of the paper, the author touches on arguments that were eventually used to produce inflation theory without citations (“black-body radiation...time required for cavity radiation to attain a desired degree of homogeneity...this will even with modest demands take much longer than its age”). These are valid arguments that have been studied earlier. While inflation theory has problems of it's own, one cannot argue against these models based on straw man arguments.

 Author’s response: It is futile to consider whether the cosmic inflation theory (Guth, 1981) might solve the homogeneity problem, because the process this theory postulates is terminated long before recombination. In the present article, the homogeneity at the stage of recombination in a Big Bang universe is not put into question. Instead, it is pointed out that homogeneity will be lost *thereafter*, irrespective of anything that might happen before. (New 3 ^rd^ passage under *Discussion*.)

 Reviewer’s comment: A previous reviewer (I. Banik) correctly identified the problem with the author's argument. The reviewer's comment “...what would be more useful is to draw two more multiple orange last scattering surface (LSS) cones translated along x,...” precisely matches my understanding of the Big Bang model. For example in Ryden's book, Fig. 9.3 ^1^ proves that the standard model considers that there is matter beyond the LSS. This is even what the author tries to describe in the paragraph starting with “In a model that is slightly less obviously untenable...” but the idea is abandoned.

 Author’s response: Fig. 9.3 in Ryden’s book (Fig. 8.4 in the 2017 edition) does not depict the Big Bang model. It depicts the supplementary Expanding View model, which is typically introduced by a figure like this. This supplementary model is meant to bring our actual observations into agreement with the theoretical expectations of a Big Bang model. This is attempted by turning the Big Bang universe inside out. This results in the ring-shaped LSS shown in Ryden’s Fig. 8.4, which corresponds to the two peripheral short red horizontal dashes in my Figure 1. Their location is spatially most remote from the LSS in the unsupplemented Big Bang model (the red horizontal dash close to the origin) by which the properties of the CMB are still derived. Standard cosmology operates, thus, with two drastically different locations of the same last scattering event, and this is irrational. (Approximately this is now said)

 By the way, simply turning the Big Bang model inside out does not invalidate the initial statements under “ *The problem*”. Even if this is done, which is a drastic error, it still needs to be considered that light propagates from the LSS faster than the constituent matter of an observer can have moved. This precludes a common place of origin for matter and the CMB  also at the periphery of the visible universe. (Now made explicit.)

 It is not either possible to replace the Big Bang model by the Expanding View model, because the latter does not predict the properties of the CMB based on its own premises – not even its very existence and that of a cosmic redshift.
